# Global burden and cross-country inequalities in diseases associated with high body mass index from 1990 to 2019: Result from the Global Burden of Disease Study 2019

**DOI:** 10.7189/jogh.14.04200

**Published:** 2024-11-08

**Authors:** Ping Wang, Shu Huang, Ruiyu Wang, Xiaomin Shi, Huan Xu, Jieyu Peng, Qi Chen, Wei Zhang, Lei Shi, Xian Zhou, Xiaowei Tang

**Affiliations:** 1 Department of Gastroenterology, the Affiliated Hospital of Southwest Medical University, Luzhou, China; 2 Nuclear Medicine and Molecular Imaging Key Laboratory of Sichuan Province, Luzhou, China; 3 Department of Gastroenterology, Lianshui County People’s Hospital, Huaian, China; 4 Department of Gastroenterology, Lianshui People’s Hospital of Kangda College Affiliated to Nanjing Medical University, Huaian, China

## Abstract

**Background:**

High body mass index (BMI) has gradually become an increased risk factor for the global burden of diseases (GBD). As the disease burden and the number of elders globally increase, it is crucial for policymakers to realise the associations between high BMI and disease burden worldwide in a timely manner and to develop effective interventions for different countries and ages.

**Methods:**

We used the GBD 2019 database to analyse the deaths and disability-adjusted life-years (DALYs) in the disease burden associated with high BMI and indicated the health inequality at the global, regional, and national levels. We applied the slope index of inequality and concentration index, two standard metrics of absolute and relative gradient inequality recommended by the World Health Organization (WHO), to quantify the distributive inequalities in the burden of diseases associated with high BMI. These rates were reported per 100 000 population as crude incidence rates, death rates, and DALYs rates. All the estimates were generated with a 95% uncertainty interval (UIs).

**Results:**

Globally, we revealed that an estimated age-standardised mortality rate associated with high BMI is 6.26 million (95% UIs = 3.99, 8.91). The age-standardised DALYs rate is 19.32 million (95% UIs = 12.77, 26.40), and the global population attributable fraction was 9% (95% UIs = 5, 12) in 2019. The largest number of high-BMI-related deaths in women mainly concentrated in the age group of 65–79 years, whereas the largest number in men was in the age group of 60–69 years. The age-standardised DALYs rate of diseases associated with high BMI was larger in the high-middle and middle socio-demographic index (SDI) (population attributable fraction (PAF) = 11 and PAF = 9) regions than those with high SDI (PAF = 1) and low SDI (PAF = 5) regions.

**Conclusions:**

In this study, our results showed that the disease burden of global deaths and DALYs associated with high BMI has substantially increased between 1990–2019. Furthermore, we demonstrated that countries with higher SDI development levels shoulders higher burden of diseases associated with high BMI. Future policies to prevent and reduce the burden should be developed and implemented based on country-specific development status.

High body mass index (BMI) (≥25 kg/m^2^) is a lifestyle-related risk factor for premature death, which is associated with various chronic diseases such as cardiovascular disease, type two diabetes mellitus, stroke, and certain types of cancer [1,2]. With the obesity population increasing to approximately 40% worldwide, the direct cost of treatment of diseases associated with high BMI has been an increasing proportion of total health care costs, especially the costs associated with the treatment of neoplastic diseases [3,4].

Recent studies showed that the deaths and disability-adjusted life-years (DALYs) associated with high BMI had more than doubled in both sexes from 1990 to 2019 [2]. Moreover, it is noted that the global deaths and DALYs associated with high BMI were higher in women than in men aged >70 years [2,5]. In 2017, the leading cause of high BMI-related DALYs was cardiovascular disease, followed by diabetes, kidney diseases, and neoplasms. The differences in socio-demographic index (SDI) levels may have contributed to the variations in high BMI-related disease burden at the Global Burden of Disease Study 2019 (GBD) at regions and country levels [6].

Obesity represents an urgent issue that should be reasonably addressed, especially in some higher disease burdens associated with high BMI regions and countries. An effective policy to eliminate the high BMI-related disease burden depends on quantitating disease burden information in a timely manner and understanding the inequality of health across countries [7–9]. However, the recent trend of the disease burden associated with high BMI remains unknown and should be specifically estimated to better address this problem.

The GBD 2019 database estimates over 369 diseases and injuries and 87 risk factors in 204 countries and territories from 1990 to 2019 [9]. Up-to-date estimates of disease deaths and burdens related to high BMI are presented based on GBD 2019, and we describe high BMI-related, spatial-temporal variations of disease burden at global, regional, and national levels by age, sex, and SDI. Thus, we can provide reasonable evidence to reveal health inequality across countries by SDI, establish related policies, programs, and measures, and reduce disease gaps between lower and higher SDI regions.

## METHODS

### Data source

In this study, the subjects were patients diagnosed with varied diseases associated with high BMI. We collected all data from GBD 2019 and aimed to analyse the global disease burden associated with high BMI from 1990 to 2019 by age group and sex, as well as to analyse the health inequality and decomposition across countries and regions. The GBD 2019 provides comprehensive and global assessments of 369 diseases and injuries, 286 causes of mortality, and 87 risk factors in 204 countries and territories, following the detailed methods used in previous editions [10,11]. For the GBD 2019, diseases and injuries are defined into four levels, ranging from the three broadest terms at level one to the most specific terms at level four. The GBD used Bradford Hill’s standard of causality and the World Cancer Research Fund’s evidence grading standard to systematically estimate epidemiological evidence supporting a causal relationship between high BMI and various disease endpoints in adults and children [12]. Death related to high BMI was identified to be correlated with six causes in grade two etiologic in global in GBD 2019, while DALYs were related with eight causes. In grade three etiologic, the numbers of causes related to high BMI resulting in death burden in women and men were 22 and 19, respectively. The number of causes contributing to the DALY burden in women and men was 26 and 23, respectively (Table S1 in the **Online Supplementary Document**).

The GBD-relevant data were sourced from censuses, household surveys, civil registrations and vital statistics, disease notifications, disease registries, health service uses, air pollution monitors, satellite images, and other sources [10]. Data on the burden of disease associated with high BMI were acquired by the Global Health Data Exchange GBD Results Tools [2]. Using a uniform methodology and standardised statistical analysis model, the GBD database provides comparable and consistent data, released every two years. The Washington University Institutional Review Committee approved the informed consent waiver for this study since de-identification and aggregation data were used in GBD 2019. This study complied with the Guidelines for Accurate and Transparent Health Estimates Reporting (GATHER) recommendations [13].

### Definitions

We included nationally or subrationally representative studies providing data on mean BMI or prevalence of overweight or obesity among adults or children. For adults, we included studies that defined overweight as BMI≥25 kg/m^2^ and obesity as BMI≥30 kg/m^2^ or if estimates using those cutoffs could be back-calculated from reported categories. For children (aged two to 18 years), we included studies that used International Obesity Task Force standards to define overweight and obesity thresholds [10]. The previous studies provided detailed methods for the source of data associated with high BMI and the process of data selection and input [10,14]. Cause-specific deaths and DALYs associated with high BMI by age, sex, years, and locations were collected by the GBD 2019 study. Estimates were given 95% uncertain intervals (UIs), defined by the 25th and the 95th values of the 1000 values, after arranging them in an ascending order using Ersatz, version 1.3 (EpiGear International, Sunrise Beach, Queensland, Australia) [14].

The burden of disease was assessed by deaths and DALYs. The DALY is a summary measure that quantifies the overall burden of diseases, which is calculated by the sum of years of life lost due to premature deaths (YLLs) and years lived with disability (YLDs) due to high BMI. One DALY can be considered as the loss of one year in complete health. The modelling methods for estimating cause-specific deaths and DALYs have been detailed elsewhere [14]. Furthermore, the SDI is a comprehensive indicator to reflect a geographical location’s development situations, which was calculated by total fertility rates among women aged <25 years, averaging the educational attainment for individuals aged >15 years and lag distributed income per capita. SDI ranges from zero to one, where zero indicates the lowest level of development, and one indicates the highest level of development. Based on the SDI quintiles, the 204 countries and territories were divided into five groups: high SDI (>0.81), high-middle SDI (0.70–0.81), middle SDI (0.61–0.69), low-middle SDI (0.46–0.60), and low SDI (<0.46).

### Population attributable fraction

The theoretical minimum risk exposure level (TMREL) indicates the level of exposure associated with the lowest risk. The TMREL of high BMI was defined based on the high BMI and was related to the lowest risk factors of all-cause mortality in future cohort studies [15]. The population attributable fraction (PAF) is a measure to estimate the reductions of a determined health metric, such as the numbers and percents of deaths or DALYs in a specific case situation, where TMREL could be compared to the changed levels of the same risk factor, by the PAF formula for a persistent risk factor [16]. We estimated PAFs using relative risk (RR) estimates from the meta-regression models and the GBD 2019 age-sex-location-year-specific estimates of the DALYs of each clinical condition included. We used the following formula [17]:



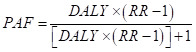



The RR is for those with the marker, as well as those who do not exhibit the marker as the referent. We then multiplied PAFs by the corresponding age-sex-location-year-specific estimates of dementia DALYs from GBD to estimate the number of dementia DALYs associated with each disease. Furthermore, the methods included four crucial information in the calculation of the burden associated with high BMI: 1) the metric of disease burden being estimated (the number of deaths, YLLs, YLDs, or DALYs, 2) the exposure levels for a risk factor (high BMI), 3) the relevant risk of a given result due to exposure, and 4) the unconditional level of risk factor exposure [18].

### Statistical analysis

We analysed age-standardised mortality rates (ASMR) and DALYs rates (ASDR) and its 95% UIs for adult populations with high BMI from 1990 to 2019 by sex, age, and region. We used the global age structure from GBD 2019 to normalise age-standardised rates (ASRs). We used population-attributable fractions to assess the ASMRs and ASDRs related to high BMI. ASRs eliminate the confounding effect of differences in age structure in the population being compared using the following formula:



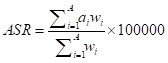



where *a_i_* represents the age-specific rate in the specific age group, *w_i_* represents the number or weight of individuals in the same specific age group from the chosen standard population, and A represents the total number of age groups. We used percentage change to show the magnitude of the change in YLDs, YLLs, DALYs and PAF from 1990 to 2019. We analysed the proportion of death and DALY rates that could be associated with high BMI in 2019 for all causes (including the level two and level three catalogue of the GBD study) by age, sex, and region.

All modelling was based on the Bayesian meta-regression model DisMod-MR, version 2.1 (WHO, Geneva, Switzerland) [19]. The GBD comparative risk assessment was divided into six key processes for each risk-outcome estimation. First, we identified convincing or probable evidence for causal high BMI-related diseases based on systematic reviews and meta-regression. Second, we estimated the RR for each risk-outcome pair as a risk-attributable function of high BMI-related diseases using GBD’s meta-regression-Bayesian, regularised, and trimmed methods. We used a Bayesian meta-regression method to generate the most prevalence and incidence estimates for each combination of sex, age, location, and year. Third, we analysed the model risk exposure level and distribution by age, sex, location, and year by using Bayesian meta-regression modelling. Further, we determined the theoretical minimum risk exposure level based on a counterfactual scenario (a given population receives the optimal level of risk exposure) and the 85th percentile of exposure in cohorts and trial studies. Further, we calculated the population-attributable fractions for each risk-outcome pair by age, sex, location, and year. Finally, we estimated the high BMI-related disease burden, wherein deaths and DALYs were multiplied by the corresponding risk factor population attributable fraction.

In terms of cross-country inequality analysis, different levels of development can be identified by stratifying the SDI values of 204 countries. Thus, using SDI to reflect the differences in the burden of high BMI-related diseases across various socioeconomic backgrounds provides a basis for cross-country health inequality analysis. The slope index of inequality and the concentration index are standardised indicators for measuring absolute and relative gradient inequalities, respectively. We calculated the slope index of inequality by regressing national DALYs rates in all ages population on an SDI-associated relative position scale, which was defined by the midpoint of the cumulative range of population ranked by SDI [20]. We examined heteroscedasticity using a weighted regression model. A negative value indicated that the indicator was more prevalent in the lowest-status subgroup. There was no difference in mortality when the index was zero. The concentration index was calculated by numerically combining the area based on the Lorenz concentration curve, which is defined as the cumulative fraction of DALYs and cumulative relative distribution of population ranked by SDI.

We also analysed the decomposition of the percentage changes (from 1990 to 2019) in deaths, DALYs, YLLs, and YLDs attributed to the high BMI observed in four factors: 1) population growth, 2) population age structure, 3) risk-related deaths, DALY, YLLs, and YLDs rates, and 4) high BMI exposure [18,19,21]. The decomposition analysis was applied by a validated algorithm that fully considered the interaction of the four factors and excluded the choice of reference year or decomposition order. The algorithm better calculates reasonable and systematic results than other existing methods [22,23].

### Uncertainty intervals

For each estimated rate and number of YLDs, YLLs, DALYs, and PAF of risk-attributable DALYs due to high BMI, we reported its 95% UI. The calculation of 95% UIs was based on 1000 draws from the posterior distribution of each stage in the estimation process using the 2.5th and 97.5th percentiles of the 1000 ordered values.

## RESULTS

### Global burden of diseases associated with high BMI

We revealed an estimated ASMR associated with high BMI was 6.26 million (95% UI = 3.992, 8.913). ASDR was 19.32 million (95% UI = 12.766, 26.397), and the global PAF was 9% (95% UI = 5, 12) in 2019. By sex, the age-standardised PAFs in men were lower than in women, but the percentage change from 1990 to 2019 in men was more than in women (**Table 1**). PAFs also varied greatly by disease type, ranging from 2% for digestive and chronic respiratory diseases to 41% for diabetes mellitus type two in both sexes in 2019. The highest change percentage of mortality PAFs associated with high BMI increased by 119% (95% UI = 68, 274) from 1990 to 2019 for liver cancer due to hepatic B (**Table 2**).

**Table 1:** Global deaths and DALYs associated with high BMI for all causes in 21 regions and SDI regions in 2019 and percentage change from 1990 to 2019

	Deaths				DALYs			
**Cause of death or DALYs**	**2019 ASR per 100 000 people, n (95% UI)**	**Change in ASR 1990–2019, % (95% UI)**	**2019 age-standardised PAF, % (95% UI)**	**Change in age-standardised PAF 1990–2019, % (95% UI)**	**2019 ASR per 100 000 people, n (95% UI)**	**Change in ASR 1990-2019, % (95% UI)**	**2019 age-standardised PAF, % (95% UI)**	**Change in age-standardised PAF 1990–2019, % (95% UI)**
Global	62.59 (39.92, 89.13)	0.05 (–0.07, 0.25)	0.09 (0.05, 0.12)	0.59 (0.42, 0.87)	1932.54 (1276.61, 2639.74)	0.18 (0.02, 0.42)	0.0589 (0.0389, 0.0801)	0.80 (0.56, 1.17)
Gender								
*Male*	66.55 (39.76, 97.21)	0.14 (–0.02, 0.44)	0.76 (0.04, 0.11)	0.71 (0.49, 1.11)	2070.34 (1311.91, 288.82)	0.27 (0.06, 0.61)	0.06 (0.03, 0.08)	0.92 (0.64, 1.42)
*Female*	58.14 (38.52, 81.38)	–0.01 (–0.12, 0.14)	0.09 (0.06, 0.13)	0.52 (0.37, 0.77)	1789.67 (1228.72, 2417.11)	0.11 (–0.02, 0.34)	0.05 (0.04, 0.07)	0.69 (0.50, 1.00)
SDI								
*High*	45.65 (29.76, 63.76)	–0.25 (–0.32, –0.12)	0.1 (0.07, 0.14)	0.14 (0.04, 0.33)	1631.11 (1120.62, 2198.13)	–0.07 (–0.18, 0.10)	0.0769 (0.0535, 0.1007)	0.18 (0.07, 0.39)
*High-middle*	69.14 (44.00, 98.24)	–0.16 (–0.23, –0.06)	0.11 (0.07, 0.16)	0.33 (0.23, 0.49)	1981.83 (1312.04, 2705.52)	–0.09 (–0.18, 0.03)	0.0828 (0.0549, 0.1123)	0.39 (0.28, 0.57)
*Middle*	68.92 (43.02, 99.26)	0.40 (0.13, 0.88)	0.09 (0.06, 0.14)	1.1 (0.74, 1.78)	2118.58 (1387.97, 2920.07)	0.48 (0.19, 0.99)	0.0737 (0.0488, 0.1008)	1.31 (0.88, 2.07)
*Low-middle*	60.34 (36.27, 88.37)	0.85 (0.39, 1.88)	0.06 (0.04, 0.09)	1.87 (1.2, 3.41)	1892.2 (1174.34, 2681.69)	1.00 (0.49, 2.08)	0.048 (0.0301, 0.0677)	2.29 (1.48, 4.02)
*Low*	55.55 (31.38, 85.09)	0.48 (0.18, 1.16)	0.05 (0.03, 0.07)	1.34 (0.89, 2.35)	1698.14 (989.56, 2491.86)	0.55 (0.22, 1.26)	0.0345 (0.0204, 0.0505)	1.65 (1.10, 2.83)
Africa								
*Southern sub-Saharan Africa*	133.84 (99.40, 171.54)	0.40 (0.25, 0.58)	0.11 (0.08, 0.14)	0.33 (0.21, 0.5)	3593.15 (2763.11, 4492.4)	0.28 (0.16, 0.44)	0.0682 (0.0521, 0.0845)	0.32 (0.20, 0.48)
*North Africa and the Middle East*	133.59 (90.04, 179.03)	0.05 (–0.09, 0.26)	0.17 (0.12, 0.23)	0.56 (0.39, 0.83)	3777.18 (2692.63, 4943.28)	0.08 (–0.06,0.29)	0.1214 (0.0872, 0.1554)	0.72 (0.53, 1.01)
*Western sub-Saharan Africa*	70.38 (43.10, 102.17)	0.65 (0.26, 1.50)	0.06 (0.04, 0.08)	1.31 (0.80, 2.38)	2033.71 (1313.87, 2877.81)	0.64 (0.26, 1.47)	0.038 (0.0246, 0.0526)	1.52 (0.96, 2.74)
*Central sub-Saharan Africa*	65.63 (34.38, 103.82)	0.02 (–0.19, 0.38)	0.05 (0.03, 0.08)	0.52 (0.33, 0.88)	1921.75 (1069.78, 2910.18)	0.05 (–0.16, 0.42)	0.0374 (0.0216, 0.0546)	0.78 (0.53, 1.22)
*Eastern sub-Saharan Africa*	59.69 (33.81, 91.86)	0.60 (0.19, 1.61)	0.05 (0.03, 0.08)	1.70 (1.01, 3.29)	1727.08 (1031.19, 2506.88)	0.58 (0.18, 1.57)	0.0359 (0.0217, 0.0517)	1.99 (1.22, 3.81)
America								
*Central Latin America*	88.47 (56.99, 122.91)	0.17 (–0.01, 0.42)	0.14 (0.09, 0.19)	0.61 (0.46, 0.9)	2918.43 (1979.02, 3924.53)	0.21 (0.06, 0.43)	0.1067 (0.0735, 0.138)	0.67 (0.51, 0.93)
*Caribbean*	84.78 (53.68, 120.24)	0.07 (–0.11, 0.32)	0.11 (0.07, 0.15)	0.34 (0.21, 0.56)	2816.65 (1904.75, 3796.92)	0.15 (–0.03, 0.38)	0.0808 (0.0559, 0.1048)	0.42 (0.29, 0.65)
*Tropical Latin America*	76.43 (53.18, 102.57)	–0.09 (–0.22, 0.17)	0.12 (0.08, 0.16)	0.45 (0.25, 0.86)	2409.66 (1731.42, 3128.1)	–0.06 (–0.19, 0.20)	0.0822 (0.0594, 0.1063)	0.46 (0.26, 0.85)
*Andean Latin America*	66.49 (42.5, 93.25)	0.13 (–0.11, 0.54)	0.12 (0.08, 0.16)	0.82 (0.57, 1.32)	2050.41 (1426.75, 2780.16)	0.13 (–0.08, 0.46)	0.08 (0.0557, 0.1035)	0.93 (0.68, 1.38)
*High-income North America*	65.58 (43.74, 86.71)	–0.08 (–0.17, 0.07)	0.13 (0.08, 0.17)	0.19 (0.07, 0.39)	2374.41 (1669.09, 3051.57)	0.05 (–0.05, 0.23)	0.0936 (0.067, 0.1172)	0.19 (0.08, 0.39)
*Southern Latin America*	64.15 (39.68, 90.84)	–0.09 (–0.22, 0.18)	0.11 (0.07, 0.15)	0.24 (0.07, 0.60)	1886.26 (1215.14, 2574.67)	–0.03 (–0.16, 0.26)	0.0773 (0.0500, 0.1045)	0.28 (0.12, 0.65)
Asia								
*Central Asia*	163.15 (107.72, 223.58)	0.37 (0.22, 0.60)	0.16 (0.11, 0.22)	0.42 (0.31, 0.60)	4303.56 (2988.64, 5696.91)	0.32 (0.18, 0.52)	0.1247 (0.0862, 0.1644)	0.63 (0.50, 0.85)
*Southeast Asia*	66.46 (39.68, 97.55)	1.22 (0.56, 3.01)	0.08 (0.05, 0.12)	2.13 (1.26, 4.60)	2201.98 (1382.81, 3086.51)	1.25 (0.62, 2.94)	0.07 (0.0445, 0.0974)	2.51 (1.54, 5.1)
*South Asia*	52.79 (30.93, 77.93)	1.13 (0.48, 2.83)	0.06 (0.03, 0.08)	2.59 (1.57, 5.31)	1729.74 (1043.52, 2440.57)	1.33 (0.62, 3.07)	0.0448 (0.0274, 0.0632)	2.98 (1.81, 5.82)
*East Asia*	40.27 (17.44, 70.19)	0.34 (–0.03, 1.61)	0.06 (0.03, 0.11)	1.36 (0.79, 3.50)	1226.16 (574.62, 2015.61)	0.42 (0.02, 1.87)	0.0549 (0.0267, 0.0885)	1.61 (0.93, 4.12)
*High-income Asia Pacific*	14.74 (6.04, 26.21)	–0.41 (–0.48, –0.25)	0.04 (0.02, 0.08)	0.05 (–0.07, 0.34)	576.33 (255.45, 972.41)	–0.26 (–0.35, –0.05)	0.0357 (0.0159, 0.0599)	0.07 (–0.05, 0.36)
Europe								
*Eastern Europe*	129.02 (85.13, 176.1)	0.08 (–0.06, 0.25)	0.15 (0.10, 0.21)	0.29 (0.16, 0.45)	3406.85 (2336.42, 4514.5)	0.10 (–0.04, 0.27)	0.1073 (0.0743, 0.1418)	0.29 (0.16, 0.45)
*Central Europe*	98.53 (65.98, 134.95)	–0.26 (–0.35, –0.13)	0.15 (0.10, 0.20)	0.14 (0.06, 0.26)	2771.08 (1976.62, 3637.96)	–0.22 (–0.31, –0.10)	0.1157 (0.0815, 0.1494)	0.15 (0.06, 0.27)
*Western Europe*	39.46 (24.17, 57.43)	–0.35 (–0.40, –0.26)	0.09 (0.06, 0.14)	0.05 (–0.04, 0.18)	1258.67 (793.46, 1767.75)	–0.23 (–0.31, –0.12)	0.0654 (0.0426, 0.0895)	0.03 (–0.05, 0.18)
Australia								
*Oceania*	139.13 (78.65, 217.90)	0.22 (0.00, 0.55)	0.11 (0.06, 0.17)	0.32 (0.19, 0.57)	4643.33 (2835.66, 6902.6)	0.26 (0.040, 0.56)	0.0941 (0.0584, 0.1341)	0.38 (0.24, 0.64)
*Australasia*	42.14 (27.07, 58.36)	–0.36 (–0.43, –0.22)	0.11 (0.07, 0.15)	0.05 (–0.06, 0.27)	1390.24 (947.98, 1867.33)	–0.23 (–0.32, –0.08)	0.0704 (0.0485, 0.0921)	0.02 (–0.08, 0.21)

ASR – age-standardised rate, DALY – disability-adjusted life year, PAF – population attributable fraction, SDI – social demographic index, UI – uncertainty interval

**Table 2:** Global deaths and DALYs associated with high BMI for both sexes combined in 2019 and percentage change from 1990 to 2019

	Deaths				DALYs			
**Cause of death or DALYs**	**2019 ASR per 100 000 people, n (95% UI)**	**Change in ASR 1990–2019, % (95% UI)**	**2019 age-standardised PAF, % (95% UI)**	**Change in age-standardised PAF 1990–2019, % (95% UI)**	**2019 ASR per 100 000 people, n (95% UI)**	**Change in ASR 1990-2019, % (95% UI)**	**2019 age-standardised PAF, % (95% UI)**	**Change in age-standardised PAF 1990–2019, % (95% UI)**
Diabetes and kidney diseases	12.54 (8.54, 17.03)	0.61 (0.41, 0.93)	0.33 (0.23, 0.45)	0.46 (0.29, 0.74)	547.26 (385.1, 728.24)	0.82 (0.57, 1.17)	0.40 (0.29, 0.51)	0.57 (0.37, 0.87)
*Diabetes mellitus*	7.57 (5.30, 9.99)	0.52 (0.33, 0.81)	0.39 (0.28, 0.51)	0.40 (0.25, 0.65)	411.06 (287.5, 552.23)	0.83 (0.59, 1.18)	0.48 (0.36, 0.59)	0.47 (0.29, 0.74)
*Diabetes mellitus type two*	7.57 (5.30, 9.99)	0.52 (0.33, 0.81)	0.41 (0.29, 0.53)	0.38 (0.22, 0.62)	411.06 (287.5, 552.23)	0.83 (0.59, 1.18)	0.51 (0.38, 0.64)	0.43 (0.26, 0.69)
*Chronic kidney disease*	4.97 (2.94, 7.36)	0.77 (0.50, 1.18)	0.27 (0.16, 0.41)	0.56 (0.35, 0.91)	136.2 (85.76, 191.33)	0.78 (0.51, 1.19)	0.26 (0.17, 0.37)	0.67 (0.43, 1.06)
*Chronic kidney disease due to diabetes mellitus type two*	1.55 (0.65, 2.60)	0.98 (0.63, 1.53)	0.30 (0.13, 0.50)	0.59 (0.34, 0.98)	40.96 (19.40, 64.76)	0.94 (0.61, 1.45)	0.34 (0.16, 0.53)	0.64 (0.39, 1.05)
*Chronic kidney disease due to glomerulonephritis*	0.67 (0.29, 1.12)	0.65 (0.42, 1.05)	0.29 (0.14, 0.47)	0.57 (0.36, 0.92)	20.87 (8.04, 34.88)	0.7 (0.44, 1.10)	0.24 (0.10, 0.39)	0.67 (0.41, 1.06)
*Chronic kidney disease due to hypertension*	1.65 (0.61, 3.00)	0.76 (0.48, 1.21)	0.28 (0.10, 0.51)	0.53 (0.31, 0.92)	38.42 (18.12, 62.01)	0.79 (0.50, 1.26)	0.31 (0.15, 0.50)	0.62 (0.37, 1.02)
*Chronic kidney disease due to other and unspecified causes*	1.09 (0.43, 1.93)	0.61 (0.36, 0.95)	0.28 (0.11, 0.47)	0.52 (0.31, 0.82)	35.95 (15.43, 58.65)	0.66 (0.43, 1.02)	0.25 (0.11, 0.40)	0.67 (0.42, 1.03)
Cardiovascular diseases	40.1 (24.72, 58.49)	–0.07 (–0.18, 0.09)	0.17 (0.10, 0.24)	0.37 (0.23, 0.60)	1045.37 (677.97, 1461.55)	–0.01 (–0.13, 0.19)	0.21 (0.14, 0.30)	0.45 (0.28, 0.74)
*Ischemic heart disease*	20.73 (12.48, 30.98)	–0.13 (–0.21, –0.01)	0.18 (0.11, 0.26)	0.26 (0.16, 0.43)	499.41 (313.30, 709.95)	–0.04 (–0.15, 0.11)	0.22 (0.14, 0.31)	0.34 (0.22, 0.55)
*Stroke*	13.20 (7.93, 19.32)	–0.09 (–0.22, 0.12)	0.16 (0.09, 0.23)	0.43 (0.26, 0.75)	416.62 (265.74, 581.11)	–0.01 (–0.16, 0.24)	0.24 (0.15, 0.32)	0.53 (0.32, 0.88)
*Intracerebral haemorrhage*	7.36 (4.42, 10.76)	0.01 (–0.16, 0.30)	0.20 (0.12, 0.30)	0.57 (0.33, 1.00)	236.21 (149.31, 329.45)	0.05 (–0.14, 0.37)	0.28 (0.18, 0.39)	0.66 (0.39, 1.13)
*Hypertensive heart disease*	5.29 (2.70, 8.58)	0.22 (–0.05, 0.58)	0.35 (0.18, 0.55)	0.55 (0.30, 0.97)	106.88 (66.08, 156.44)	0.18 (–0.05, 0.56)	0.40 (0.25, 0.56)	0.61 (0.35, 1.05)
*Ischemic stroke*	4.61 (2.50, 7.29)	–0.18 (–0.26, –0.07)	0.11 (0.06, 0.17)	0.23 (0.15, 0.37)	132.05 (80.14, 195.95)	–0.05 (–0.15, 0.10)	0.17 (0.10, 0.24)	0.33 (0.23, 0.52)
*Subarachnoid haemorrhage*	1.24 (0.79, 1.74)	–0.21 (–0.40, 0.12)	0.27 (0.18, 0.36)	0.85 (0.47, 1.55)	48.36 (32.49, 65.61)	–0.15 (–0.34, 0.16)	0.35 (0.24, 0.46)	0.84 (0.48, 1.49)
*Atrial fibrillation and flutter*	0.89 (0.48, 1.44)	0.29 (0.15, 0.49)	0.20 (0.11, 0.32)	0.26 (0.16, 0.45)	22.46 (11.86, 37.08)	0.26 (0.15, 0.44)	0.21 (0.12, 0.32)	0.30 (0.19, 0.47)
Neoplasms	5.69 (3.21, 8.83)	0.22 (0.07, 0.43)	0.05 (0.03, 0.07)	0.44 (0.28, 0.68)	133.93 (76.19, 206.81)	0.22 (0.05, 0.46)	0.04 (0.03, 0.07)	0.52 (0.33, 0.82)
*Oesophageal cancer*	1.09 (0.34, 2.10)	0.21 (–0.04, 0.63)	0.18 (0.06, 0.35)	0.62 (0.32, 1.16)	26.27 (8.12, 49.89)	0.18 (–0.08, 0.59)	0.19 (0.06, 0.36)	0.68 (0.37, 1.25)
*Colon and rectum cancer*	1.07 (0.58, 1.70)	0.24 (0.13, 0.40)	0.08 (0.04, 0.12)	0.29 (0.21, 0.45)	24.41 (13.52, 38.55)	0.28 (0.16, 0.45)	0.08 (0.05, 0.13)	0.33 (0.23, 0.50)
*Chronic respiratory diseases*	0.94 (0.51, 1.53)	–0.2 (–0.36, 0.05)	0.02 (0.01, 0.03)	0.37 (0.12, 0.79)	44.8 (26.37, 68.64)	–0.12 (–0.26, 0.09)	0.03 (0.02, 0.05)	0.43 (0.22, 0.77)
*Liver cancer*	0.74 (0.29, 1.39)	0.29 (–0.01, 0.93)	0.12 (0.05, 0.24)	0.94 (0.55, 1.88)	19.24 (7.56, 36.41)	0.21 (–0.10, 0.90)	0.13 (0.05, 0.25)	1.07 (0.62, 2.23)
*Breast cancer*	0.55 (0.23, 0.99)	0.30 (0.06, 0.91)	0.06 (0.03, 0.12)	0.48 (0.22, 1.19)	11.22 (3.53, 21.43)	0.43 (0.06, 1.69)	0.05 (0.01, 0.09)	0.59 (0.19, 1.95)
*Uterine cancer*	0.45 (0.31, 0.61)	–0.01 (–0.10, 0.11)	0.40 (0.28, 0.53)	0.29 (0.18, 0.45)	11.22 (7.71, 15.03)	0.03 (–0.08, 0.17)	0.40 (0.28, 0.53)	0.32 (0.20, 0.50)
*Pancreatic cancer*	0.40 (0.15, 0.74)	0.52 (0.36, 0.72)	0.06 (0.02, 0.11)	0.22 (0.11, 0.38)	8.54 (3.09, 15.99)	0.53 (0.37, 0.73)	0.06 (0.02, 0.12)	0.26 (0.15, 0.43)
*Kidney cancer*	0.39 (0.23, 0.59)	0.34 (0.23, 0.51)	0.19 (0.11, 0.28)	0.20 (0.12, 0.33)	9.05 (5.33, 13.42)	0.3 (0.18, 0.48)	0.18 (0.11, 0.27)	0.24 (0.14, 0.38)
*Gallbladder and biliary tract cancer*	0.33 (0.17, 0.53)	–0.06 (–0.18, 0.11)	0.15 (0.08, 0.25)	0.12 (0.01, 0.34)	6.86 (3.69, 11.17)	–0.04 (–0.17, 0.15)	0.16 (0.08, 0.25)	0.18 (0.06, 0.42)
*Liver cancer due to hepatitis B*	0.29 (0.10, 0.58)	0.13 (–0.20, 0.98)	0.12 (0.04, 0.25)	1.19 (0.68, 2.74)	8.86 (3.15, 17.47)	0.08 (–0.26, 0.93)	0.13 (0.05, 0.26)	1.27 (0.71, 2.95)
*Leukaemia*	0.27 (0.13, 0.46)	0.12 (0.01, 0.32)	0.06 (0.03, 0.11)	0.53 (0.34, 0.83)	7.10 (3.49, 12.09)	0.12 (–0.01, 0.35)	0.05 (0.02, 0.08)	0.78 (0.48, 1.27)
*Liver cancer due to hepatitis C*	0.24 (0.09, 0.44)	0.36 (0.12, 0.89)	0.13 (0.05, 0.24)	0.72 (0.43, 1.34)	5.06 (2.03, 9.24)	0.29 (0.03, 0.84)	0.14 (0.06, 0.26)	0.83 (0.50, 1.56)
*Liver cancer due to alcohol use*	0.18 (0.07, 0.34)	0.55 (0.29, 0.99)	0.16 (0.06, 0.30)	0.68 (0.43, 1.19)	4.31 (1.63, 8.16)	0.50 (0.23, 0.97)	0.17 (0.07, 0.31)	0.76 (0.48, 1.34)
*Non-Hodgkin lymphoma*	0.17 (0.07, 0.30)	0.32 (0.20, 0.53)	0.05 (0.02, 0.10)	0.30 (0.20, 0.51)	4.3 (1.83, 7.66)	0.32 (0.19, 0.53)	0.05 (0.02, 0.09)	0.38 (0.25, 0.61)
*Multiple myeloma*	0.10 (0.04, 0.18)	0.34 (0.16, 0.57)	0.07 (0.03, 0.12)	0.32 (0.18, 0.53)	2.17 (0.97, 3.84)	0.33 (0.16, 0.57)	0.07 (0.03, 0.12)	0.34 (0.20, 0.57)
*Other leukaemia*	0.09 (0.04, 0.16)	0.02 (–0.13, 0.26)	0.06 (0.03, 0.10)	0.76 (0.45, 1.24)	2.33 (1.12, 4.08)	–0.02 (–0.19, 0.27)	0.05 (0.02, 0.08)	1.19 (0.67, 2.01)
*Acute myeloid leukaemia*	0.09 (0.04, 0.15)	0.43 (0.20, 0.68)	0.07 (0.04, 0.12)	0.33 (0.20, 0.53)	2.26 (1.13, 3.74)	0.38 (0.21, 0.63)	0.06 (0.03, 0.10)	0.41 (0.23, 0.69)
*Ovarian cancer*	0.08 (0.00, 0.17)	0.14 (0.01, 0.29)	0.03 (0.00, 0.07)	0.17 (0.10, 0.31)	2.00 (–0.06, 4.54)	0.17 (0.03, 0.35)	0.03 (0.00, 0.07)	0.17 (0.10, 0.34)
*Thyroid cancer*	0.06 (0.03, 0.10)	0.30 (0.13, 0.53)	0.1 (0.05, 0.17)	0.37 (0.21, 0.59)	1.54 (0.77, 2.57)	0.38 (0.20, 0.65)	0.10 (0.05, 0.17)	0.44 (0.27, 0.70)
*Chronic lymphoid leukaemia*	0.05 (0.02, 0.08)	0.15 (0.04, 0.34)	0.08 (0.04, 0.14)	0.24 (0.16, 0.40)	0.94 (0.46, 1.59)	0.17 (0.06, 0.37)	0.08 (0.04, 0.13)	0.26 (0.17, 0.41)
*Liver cancer due to other causes*	0.04 (0.01, 0.07)	0.21 (–0.09, 0.90)	0.11 (0.04, 0.21)	1.10 (0.61, 2.32)	1.01 (0.39, 1.92)	0.14 (–0.17, 0.86)	0.09 (0.04, 0.17)	1.11 (0.56, 2.54)
*Chronic myeloid leukaemia*	0.02 (0.01, 0.04)	–0.3 (–0.38, –0.17)	0.06 (0.03, 0.10)	0.3 (0.11, 0.66)	0.66 (0.33, 1.10)	–0.25, (–0.35, –0.1)	0.05 (0.03, 0.08)	0.43 (0.13, 1.11)
*Acute lymphoid leukaemia*	0.02 (0.01, 0.04)	0.33 (0.12, 0.55)	0.04 (0.02, 0.07)	0.60 (0.34, 1.11)	0.90 (0.43, 1.56)	0.44 (0.18, 0.72)	0.03 (0.01, 0.04)	0.83 (0.47, 1.57)
Neurological disorders	2.81 (0.46, 8.42)	0.27 (0.15, 0.53)	0.09 (0.03, 0.17)	0.25 (0.14, 0.52)	42.22 (11.24, 107.37)	0.30 (0.18, 0.56)	0.03 (0.01, 0.08)	0.32 (0.19, 0.59)
*Alzheimer disease and other dementias*	2.81 (0.46, 8.42)	0.27 (0.15, 0.53)	0.12 (0.05, 0.23)	0.23 (0.11, 0.47)	42.22 (11.24, 107.37)	0.30 (0.18, 0.56)	0.12 (0.05, 0.23)	0.25 (0.14, 0.50)
Digestive diseases	0.51 (0.31, 0.76)	–0.05 (–0.17, 0.12)	0.02 (0.01, 0.02)	0.38 (0.22, 0.64)	24.94 (14.72, 39.98)	0.06 (–0.07, 0.23)	0.02 (0.01, 0.04)	0.51 (0.31, 0.8)
*Gallbladder and biliary diseases*	0.51 (0.31, 0.76)	–0.05 (–0.17, 0.12)	0.31 (0.19, 0.44)	0.38 (0.22, 0.62)	24.94 (14.72, 39.98)	0.06 (–0.07, 0.23)	0.32 (0.20, 0.45)	0.44 (0.29, 0.68)
Asthma	0.94 (0.51, 1.53)	–0.20 (–0.36, 0.05)	0.16 (0.09, 0.25)	0.63 (0.38, 1.14)	44.8 (26.37, 68.64)	–0.12 (–0.26, 0.09)	0.16 (0.10, 0.24)	0.53 (0.31, 0.9)

ASR – age-standardised rate, DALY – disability-adjusted life year, PAF – population attributable fraction, UI – uncertainty interval

**Figure 1:**
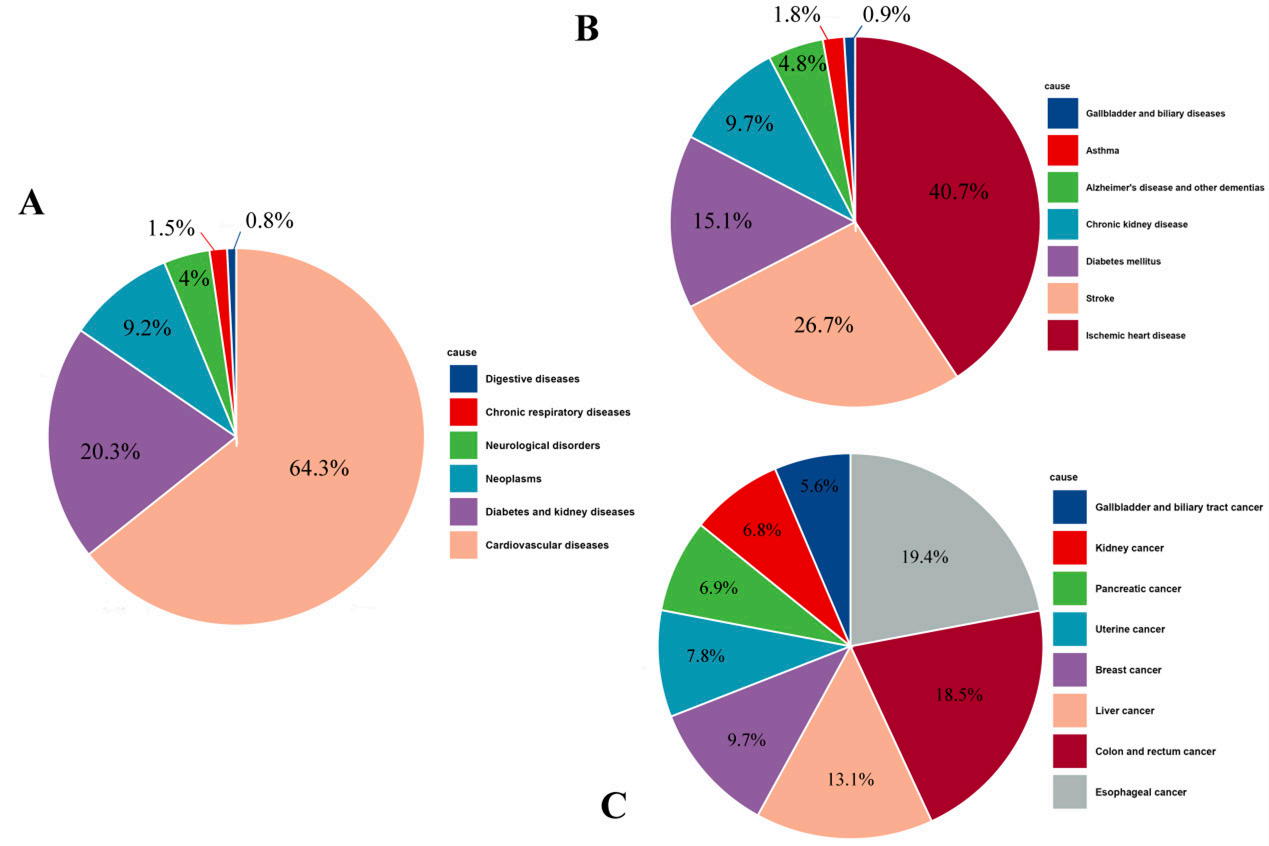
Contribution of each disease to the total burden associated with high BMI in 2019. **Panel A**. The level-two catalogue of the GBD study. **Panel B.** The level-three catalogue of GBD study except for the neoplasms. **Panel C.** The neoplasms.

Regarding the contribution of mortality numbers for each disease to the total disease burden associated with high BMI in 2019, cardiovascular diseases contributed the largest percentage (64.3%) in the level-two catalogue of the GBD study, followed by diabetes and kidney diseases (26.7%), and the neoplasms (15.1%) (**Figure 1**, Panel A). For the level-three catalogue of GBD study, except for the neoplasms, the mortality numbers associated with high BMI ranking in the top three were ischemic heart disease (40.7%), stroke (26.7%), and diabetes mellitus (15.1%) (**Figure 1**, Panel B). For the neoplasms, the highest mortality number was in oesophageal cancer (19.4%), followed by colon and rectum cancer (18.5%), and liver cancer (13.1%) (**Figure 1**, Panel C)

### Global burden of diseases associated with high BMI by age and sexes

**Figure 2:**
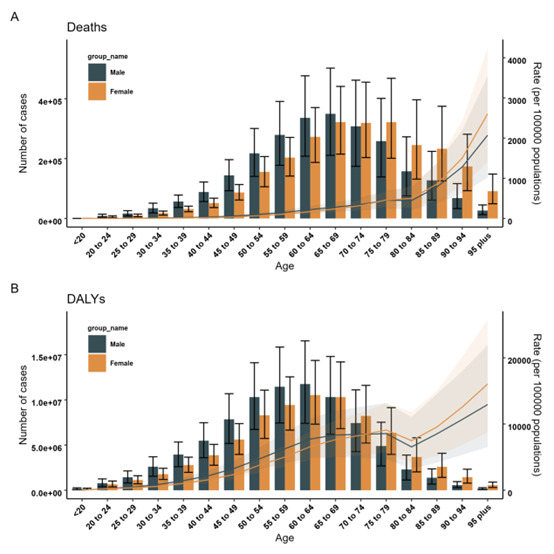
Age-specific numbers and rates of deaths and DALYs associated with high BMI by sex in 2019. **Panel A.** Deaths. **Panel B.** DALYs. DALY – disability-adjusted life year.

Age-specific rates of high BMI-related deaths and DALYs increased with age increase, except DALY in people aged 80–84 years (**Figure 2**). The largest number of high-BMI-related deaths in women mainly concentrated in the age group of 65–79 years, whereas the largest number in men was in the age group of 60–69 years. The number of high-BMI-related DALY peaked in the age group of 60–69 years in both sexes (**Figure 2**).

The age-specific numbers and rate of mortality and DALYs varied by disease and sex (Figures S1–8 in the **Online Supplementary Document**). The age-specific mortality and DALYs rates and numbers trend of cardiovascular diseases and diabetes and kidney diseases were similar to the overall diseases trend on the above (Figure S1–2 in the **Online Supplementary Document**). The trend of age-specific mortality and DALYs rates was increasing in both sexes with increasing ageing among people suffering from the neoplasm, digestive diseases, chronic respiratory diseases, neurological disorders, sense organ diseases, and musculoskeletal disorders, and the rates trend in women were always more than in men for the above diseases excepted neoplasms (Figures S3 and S8 in the **Online Supplementary Document**). The highest mortality numbers associated with high BMI were mainly concentrated on older people, such as digestive diseases and neurological disorders in the age group of 80–89 years (Figure S3 and S6 in the **Online Supplementary Document**). However, the highest age-specific DALYs numbers of digestive diseases and musculoskeletal disorders were chiefly in the middle-aged and elderly, respectively, in the age group of 50–54 and 55–59 years (Figure S3 and S6 in the **Online Supplementary Document**).

### Global burden of diseases associated with high BMI by 21 regions

**Figure 3:**
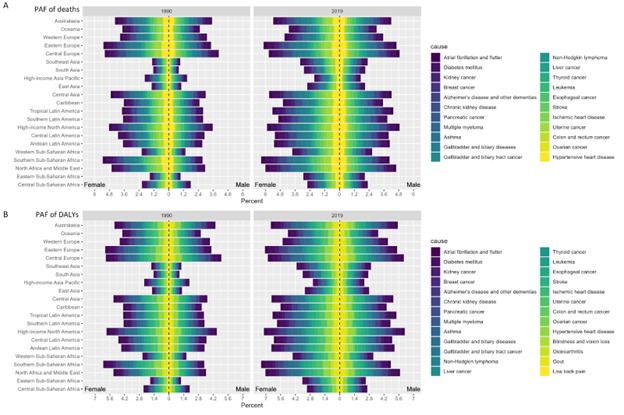
PAF of diseases for GBD level-three of deaths and DALY associated with high BMI in 21 GBD regions by sex in 1990 and 2019. **Panel A.** Deaths. **Panel B.** DALYs. DALY – disability-adjusted life year, PAF – population attributable fraction.

Region-specific estimates show that the lowest PAFs were in the high-income Asia Pacific. The percentage change of PAF from 1990 to 2019 in the high-income Asia Pacific decreased by 41% (95% UI = 25, 48) compared to the rest of the regions of Asia, especially Central Asia, which had the highest PAF among all Asia regions (**Figure 3**, **Table 1**). Southern sub-Saharan Africa, Eastern Europe, North Africa, and the Middle East were ranked the top three for the deaths and DALYs of PAFs in 2019 (**Figure 3**). Uterine cancer had the highest deaths of PAF in high-income North America, while gallbladder and biliary tract cancer had the highest deaths of PAF in Eastern Europe, and oesophageal cancer had the highest deaths of PAF in Southern sub-Saharan Africa (**Figure 3**).

The leading causes and cancers of the ASDR associated with high BMI by regions for women and men in 2019 are shown in Figure S9, Panels A and C in the **Online Supplementary Document**. The Southern sub-Saharan Africa region had the highest DALYs PAF associated with high BMI in women for diabetes mellitus (70%), oesophageal cancer (35.9%), ischemic heart disease (34.3%), and colon and rectum cancer (7.8%) (Tables S2–4 in the **Online Supplementary Document**). High-income North America had the highest DALYs PAFs in men for stroke (37.6%), oesophageal cancer (35.6%), ischemic heart disease (34.6%), liver cancer (26.4%), colon and rectum cancer (19.1% in men and 16.9% in women) (Figure S9, Panels A and C in the **Online Supplementary Document**). Furthermore, Central Europe had the highest PAFs in men for diabetes mellitus (63.0%), and Andean Latin America had the highest PAFs in women for stroke (42.1%) (Figure S9, Panel A in the **Online Supplementary Document**).

### Global burden of diseases associated with high BMI by SDI regions and cross-national health inequality analysis

The ASR of diseases-related DALYs associated with high BMI was larger in countries with high-middle and middle SDI (PAF = 11 and PAF = 9) than those with high SDI (PAF = 1) and low SDI (PAF = 5) (**Table 1****,** Figure S10 in **Online Supplementary Document**). Specially, middle and high-middle SDI regions, such as Oceania, Central Asia, and Eastern Europe had the highest ASR of all causes-related DALYs associated with high BMI. In contrast, high SDI regions, such as high-income Asia Pacific and low-middle SDI regions, such as South Asia and East Asia, had the lowest ASR of all causes-related DALYs associated with high BMI. High SDI regions such as high-income Asia Pacific, Western Europe, and Australasia showed a decline in ASR of all-causes DALYs associated with high BMI from 1990 to 2019, except the high-income North America having increasing trends. In contrast, lower and middle SDI regions such as Eastern sub-Saharan Africa, South Asia and Southeast Asia showed an increase in ASR of all causes-related DALYs attributed to high BMI (Figure S10 in the **Online Supplementary Document**).

**Figure 4:**
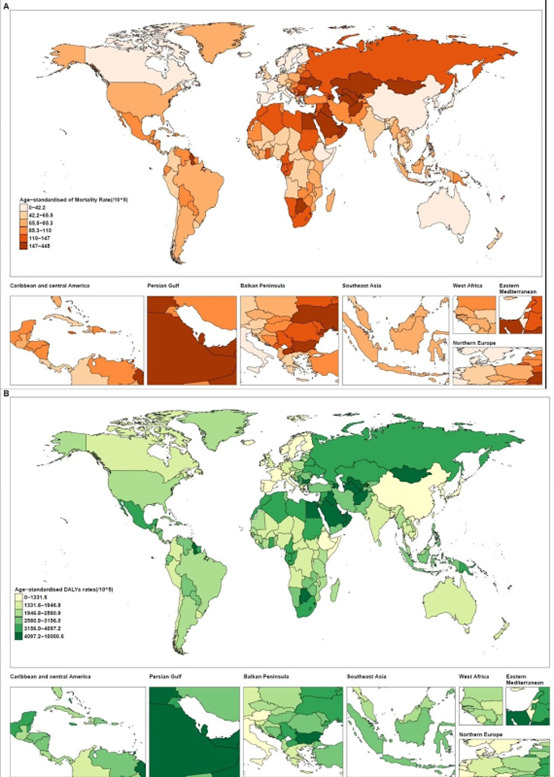
Age-standardised death and DALYs rates associated with high BMI for both sexes combined in 2019. **Panel A.** Deaths. **Panel B.** DALYs.

In 2019, age-standardised mortality and DALY rates and the estimated annual percentage change of the disease burden associated with the high BMI, country-specific in both sexes, are shown in **Figure 4** and Figure S16 in the **Online Supplementary Document**. Globally, the highest ASMR is 319 per 100 000 people in Fiji, followed by 302 in Nauru and 284 in Kiribati. The lowest age-standardised mortality and DALY rates among African regions were in Somalia and Ethiopia, while the highest rate was in Egypt and Botswana (**Figure 4**). In the men, the highest PAF of 24.4% was in the Fiji and Cook Islands, followed by 23.6% in the United Arab Emirates and 22.3% in Qatar (Figure S11 in the **Online Supplementary Document**). In women, Fiji also had the highest PAF, with 30% of all causes associated with high BMI, followed by the Cook Islands (28.2%) and Qatar (27.3%). Countries in sub-Saharan Africa, East Asia, and South Asia had consistently lower overall PAFs than those in other regions, with less than 4% in men and less than 5% in women (Figure S11 in the **Online Supplementary Document**).

**Figure 5:**
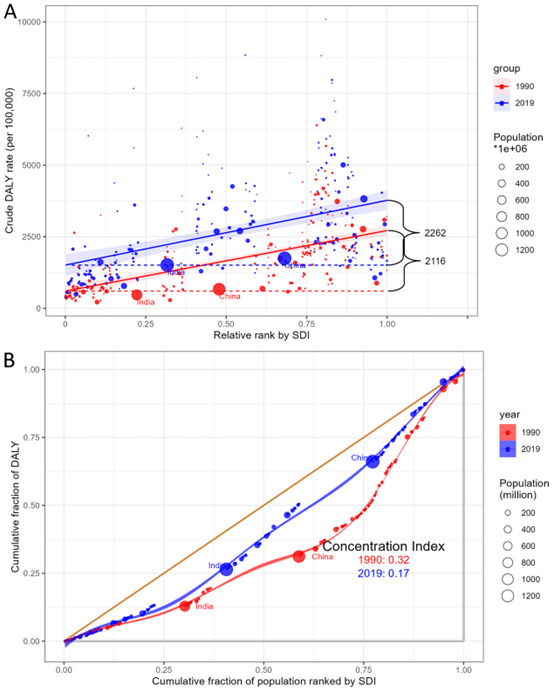
Health inequality regression and concentration curves for the DALYs of all causes in all ages worldwide, 1990 and 2019. **Panel A.** Health inequality regression curves of DALYs. **Panel B.** Concentration curve of DALYs. DALYs – disability-adjusted life-years.

Significant absolute and relative SDI-related inequalities in the burden of all diseases attributed to high BMI were observed, with a disproportionately higher burden shouldered by countries with high-middle SDI such as Kiribati, Nauru, and Fiji (**Figure 5** and Figure S12 in the **Online Supplementary Document**). As demonstrated by the slope index of inequality, the gap in DALYs rate between the highest and the lowest SDI country increased from 2116 in 1990 to 2262 in 2019. Moreover, the concentration index showed 0.32 in 1990 and 0.17 in 2019 (Figure S12 in the **Online Supplementary Document**).

### Global decomposition analysis of the change in the number of disease deaths associated with high BMI

**Figure 6:**
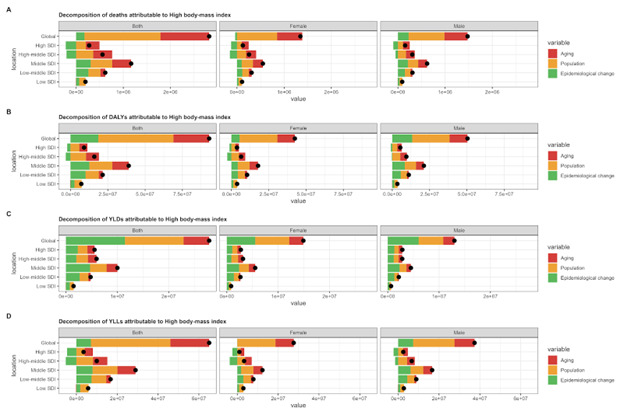
Decomposition of deaths, DALYs, YLDs, and YLLs in all causes associated with high BMI in global and SDI regions, 1990–2019. **Panel A.** Deaths. **Panel B.** DALYs. **Panel C.** YLDs. **Panel C.** YLLs. Percent change in risk-attributable deaths, DALYs, YLDs, and YLLs. Results are shown for all causes combined. The black dot shows the total percentage change. The risk-deleted DALY rate is the expected DALY rate if the exposure level for high BMI was reduced to the theoretical minimum risk exposure level. BMI – body mass index, DALYs – disability-adjusted life-years, YLDs – years lived with disability, YLLs – years of life lost.

The past 30 years have seen a remarkable global increase in deaths, DALYs, YLDs and YLLs attributed to high BMI, with the largest increase occurring in middle SDI regions (**Figure 6** and Figures S13–15 in the **Online Supplementary Document**). First, in terms of all causes-related deaths attributed to high BMI, ageing and population growth accounted for 36.6% and 57.11% in both men and women, 33.21% and 51.46% in men, 39.63% and 63.31% in women, of worldwide increase in deaths, respectively, with the most significant ageing contribution occurring in high SDI regions (104.8% in both men and women, 94.71% in men, and 120.99% in women, respectively), where the population growth had the largest effects on deaths growth (74.6% in both sexes, 69.66% in men, and 80.47% in women, respectively). The effect of epidemiological change on deaths growth was positive (6.28% in both sexes and 15.33% in men) and negative (–2.94% in women), and this effect was the most pronounced in the low-middle SDI regions (42.18% in both sexes, 36.21% in women, and 47.96% in men). On the contrary, the effect of epidemiological change on death growth associated with high BMI was the most negatively pronounced in the high SDI regions (–79.41% in both sexes, –64.37% in men and –101.46% in women) (**Figure 6**).

## DISCUSSION

Although current studies have led to a better understanding of obesity epidemiology and better management of diseases associated with high BMI, the global burden of diseases associated with high BMI has shown an upward pattern over the past decades [5]. Previous studies demonstrated that diseases associated with high BMI remain a health challenge in different regions and countries [24]. Our results highlight that the ASMR associated with high BMI is 6.26 million, the ASR of DALYs is 19.32 million, and the global PAF was 9% in 2019 worldwide. In Oceania, Fiji, Nauru, and Kiribati, the ASMR and ASR of DALYs associated with high BMI were the highest. Thus, the high BMI-related disease burden should warrant surveillance and monitoring of BMI regularly as well as the analysis and identification of the risk factor of such an increase to design and produce appropriate evidence-based public health measures and interventions and improve the current high-BMI-related disease burden.

The overall ASMR and ASDR of disease burden associated with high BMI globally was higher in men than women in 2019, and women presented a decrease in ASMR from 1990 to 2019 compared to men during the same periods. However, the global deaths and DALYs rates associated with high BMI were higher in women than men aged >70 years but lower in women than in men aged <70 years. The reasons for these results were not completely understood. Thus, the global burden of disease associated with high BMI with respect to sex difference should be recognised and implemented specific strategies, which are urgently needed along with the social structure of population ageing.

Population growth and ageing are crucial indicators of increased DALYs, deaths, YLLs, and YLDs of diseases burden associated with high BMI in the global, middle, low-middle, and low SDI regions, but current epidemiological change trends (early preventions, diagnosis and treatment of diseases associated with high BMI, thereby reducing potential morbidity) do not counteract the contribution of population growth and ageing to the increased burden of diseases associated with high BMI. Our study results showed that the DALYs rates in 2019 generally increased along with the ageing. Thus, population growth has driven the increasing DALYs burden of diseases associated with high BMI. Despite the fact that the disease burden associated with high BMI is closely related to ageing, the disease burden associated with high BMI in the elderly cannot be considered to be caused by ageing alone.

In many regions, our results showed that Southern sub-Saharan Africa, Eastern Europe, North Africa, and the Middle East were the top three ranking for the deaths and DALYs of PAFs in 2019. Although governments have put forward many interventions to improve the disease burden associated with high BMI over the past decade, the disease burden has shown an increasing trend in the low and middle SDI regions and the middle and high-middle SDI regions, such as Oceania, Central Asia, Eastern Europe had the highest ASR of all causes-related DALYs associated with high BMI [25,26]. The consistent relationships between high BMI and poor health outcomes are concerning in the middle and higher SDI regions. The increasingly sedentary lifestyles and the modernisation and globalisation of the food supply have contributed to the rising obesity problem [25]. Consequently, there is a need for more studies and attention to generate awareness and find solutions to the obesity problem.

As reported in previous studies, the leading causes of high-BMI-related death were cardiovascular disease, diabetes and kidney disease, and neoplasm [2]. Similar to the previous research, the cardiovascular diseases associated with high BMI have shown a significant upward trend in low SDI regions. However, diabetes and kidney diseases and the neoplasm have shown remarkably increasing trends in the low-middle SDI regions in the past 30 years. In some lower SDI regions and countries, higher-income people may prefer larger body sizes, obtain more fatty food, and engage in light labour [27]. In 2019, our results showed that about 42% of the ASDR of ischemic heart disease was associated with high BMI in adults aged 30–34 years, which had the highest burden among all age groups. Lipid accumulation and fatty streaks develop in many adults, with obesity accelerating atherosclerotic changes through mechanisms such as insulin resistance and inflammation. We also found that the percentage change of the ranking top three diseases such as cardiovascular diseases, diabetes and kidney diseases, and the neoplasm associated with high BMI in age-standardised PAF from 1990 to 2019 in many middle SDI regions, such as Southeast Asia and South Asia, had the dramatic increase, because dietary fats, animal products by people and longer sitting lifestyles led to significantly increase in the prevalence of high BMI [27,28]. The presence of high BMI can lead to the exacerbation of metabolic risk factors, such as dyslipidaemia, elevated blood pressure, and hyperglycaemia, all of which play a crucial role in the development and progression of various diseases in affected individuals. Furthermore, a fast food diet with higher fat and higher sugar is believed to increase blood cholesterol and prevent cardiovascular diseases and diabetes. It is gradually replacing the traditional and healthy diet of vegetables, bean products, cereals, and fish [29,30]. Unhealthy diets and lack of exercise are the main reasons for high BMI in these regions [8].

We also estimated that 5% of deaths and 4.3% of DALYs were due to cancers associated with high BMI in 2019. Previous research by GBD study showed that 3.9% of global cancer in 2012 and 9.7% of global cancer in 2017 were associated with high BMI. Most attributable cancers DALYs were accounted for by behavioural risk factors such as smoking, alcohol use, unsafe sex and dietary habits leading to high BMI, indicating a need for effective measures to address various risk factors to eliminate cancer burden globally [11]. The high BMI was ranked number three at the most detailed risk factor levels contributing to global cancer burden from 2010 to 2019 in high SDI regions [11]. Furthermore, oesophageal cancer had the highest ASDR in the Southern sub-Saharan Africa regions and the age group of 55–59 years, accounting for 36% and 21%, respectively. These findings found that the constantly growing disease burden associated with high BMI in different diseases demonstrates that weight control is important and crucial to reducing the risk of diseases associated with high BMI to a great extent [31]. Health policy and prevention measures to control weight are crucial for people who have high BMI to avoid gaining diseases associated with high BMI, and to eliminate the increasing burden of diseases associated with high BMI, emphasis should be placed on them.

The measurement of across-country SDI-related inequalities in the disease burden associated with high BMI could indicate the associations of burden distribution with the sociodemographic development levels. Our study first indicated the cross-country inequality analysis on high BMI-related disease burden according to standard health analysis methods produced by the WHO and the results showed that countries with higher SDI had a more disproportional burden of diseases associated with high BMI. However, we generally recommend that individuals who live in high SDI countries have more chances to enjoy higher quality health and medical services, thus producing a lower disease burden. The interpretation of the association between disease burden associated with high BMI and sociodemographic development levels observed in this study may be attributed to two aspects. On the one hand, lifestyle changes and the development of the economy are generally recognised to result in the more easily to access high-sugar and high-fat dietary, in which genetics only accounted for a minority of the disease burden associated with high BMI and may play a little role [32]. On the other hand, disease burdens associated with high BMI are characterised by the chronic long-term disease course and multiple disease burdens associated with high BMI in the high SDI regions, leading to a huge demand for medical care. Furthermore, the disease burden associated with high BMI overall increased in the SDI-related inequalities across 204 countries from 1990 to 2019, demonstrating that the measurement put into prevention, management, and treatment of disease burden associated with high BMI along with sociodemographic development may be insufficient over the past 30 years.

### Limitations

Our study has several limitations commonly described in previous published GBD studies [2,12]. First, although many methods, such as correcting for classification and incompleteness and redistributing garbage codes, were applied to reduce bias in estimates, the data collected from different countries and regions may have several disparities in quality, comparability, accuracy and missing degree, which may lead to a certain deviation in the estimations [15]. Data resources were dependent on the quality of each country’s vital registry, and modelling was applied to data that was lacking, potentially introducing bias into estimates. In some low SDI regions and countries where there was poor access to care and diagnostics, there might also have been substantial underreporting and underdiagnosis of diseases associated with high BMI. The GBD study employed a different modelling system to estimate the burden in countries with no data available, meaning that the results are based on estimations rather than real data. Second, the GBD 2019 defined a high BMI in adults as a BMI of 20–25 kg/m2, and we recognised that the cut-point for high BMI varied by race and ethnicity [15,33]. The self-reported estimates of high BMI are not reliable. Women in the US vs women in the Arab countries will either underestimate or overestimate their true weight, on average. Furthermore, sensitivity analysis unitising the established in different sociographical regions and countries could not be accurately calculated due to a paucity of data in GBD 2019, which has resulted in an underestimation of the burden of disease associated with high BMI, especially in Asian countries. The specific context in each country may cause locally specific combinations that may be difficult to encompass in a single analysis, even worse for the within-country regions.

## CONCLUSIONS

Our results showed that the disease burden of global deaths and DALYs associated with high BMI substantially increased between 1990–2019. Furthermore, we demonstrated that countries with higher SDI development levels shoulder a higher burden of diseases associated with high BMI and the magnitude of the sociodemographic development level-related inequalities exacerbated over the past 30 years, indicating another line of evidence on the model of burden distribution across sociodemographic development levels in the disease burden associated with high BMI. Successful population-wide activities targeting excessive weight and high BMI may alleviate the burden of various diseases. Because of the large variations in the high-BMI-related burden of disease by SDI, future policies to prevent and reduce the burden should be developed and implemented based on country-specific development status.

## Additional material


Online Supplementary Document
