# Supplementary material for: Global burden and cross-country inequalities in diseases associated with high body mass index from 1990 to 2019: Result from the Global Burden of Disease Study 2019
Source: J Glob Health. 2024 Nov 8;14:04200. doi: 10.7189/jogh.14.04200 (PMC11544517; doi:10.7189/jogh.14.04200)
Supplement: Online Supplementary Document [file jogh-14-04200-s001.pdf]

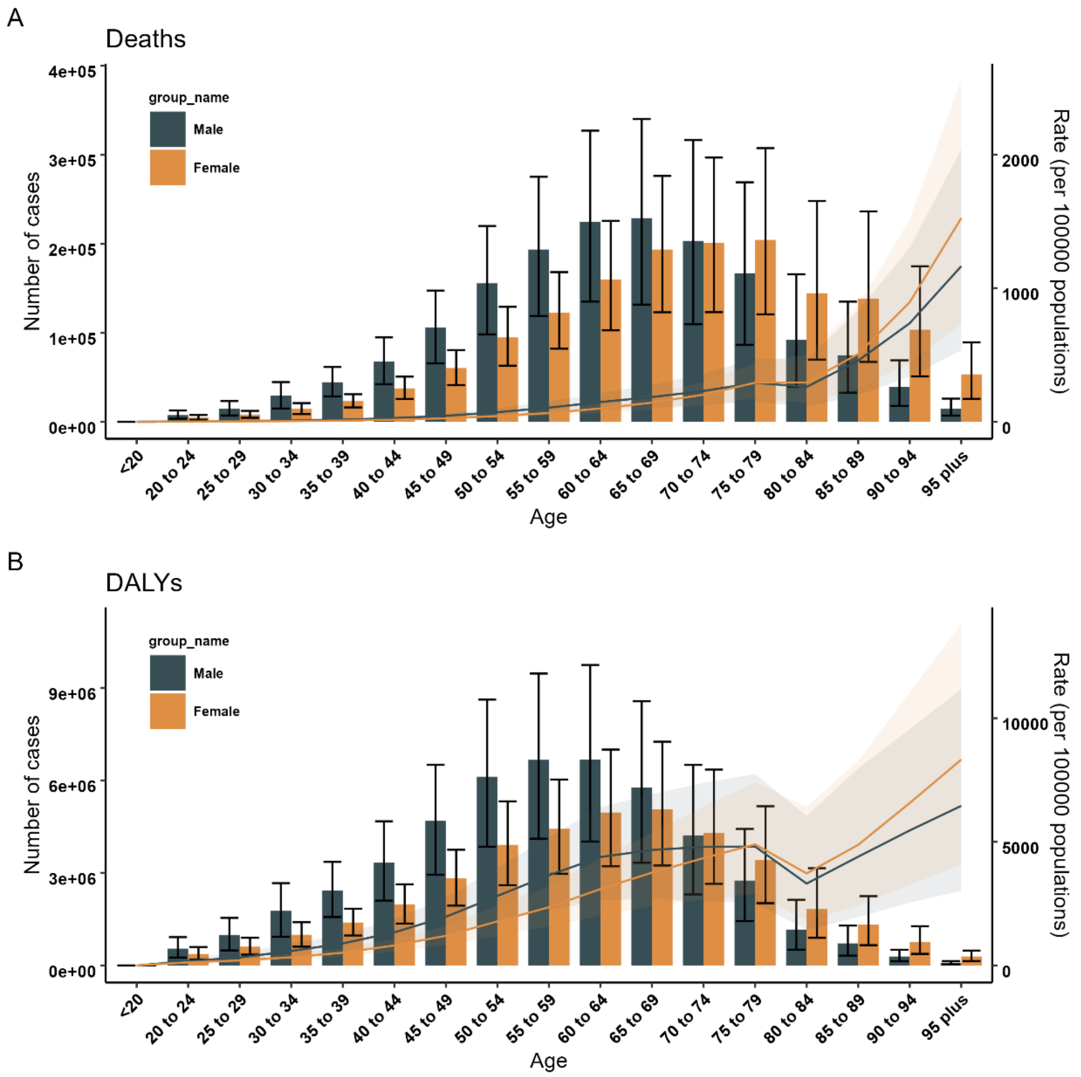

Supplementary Figure 1. Age-specific numbers and rates of deaths and DALYs for cardiovascular diseases due to high body mass index by sex, in 2019.

(A) Deaths. (B) DALYs. DALY, disability-adjusted life year.

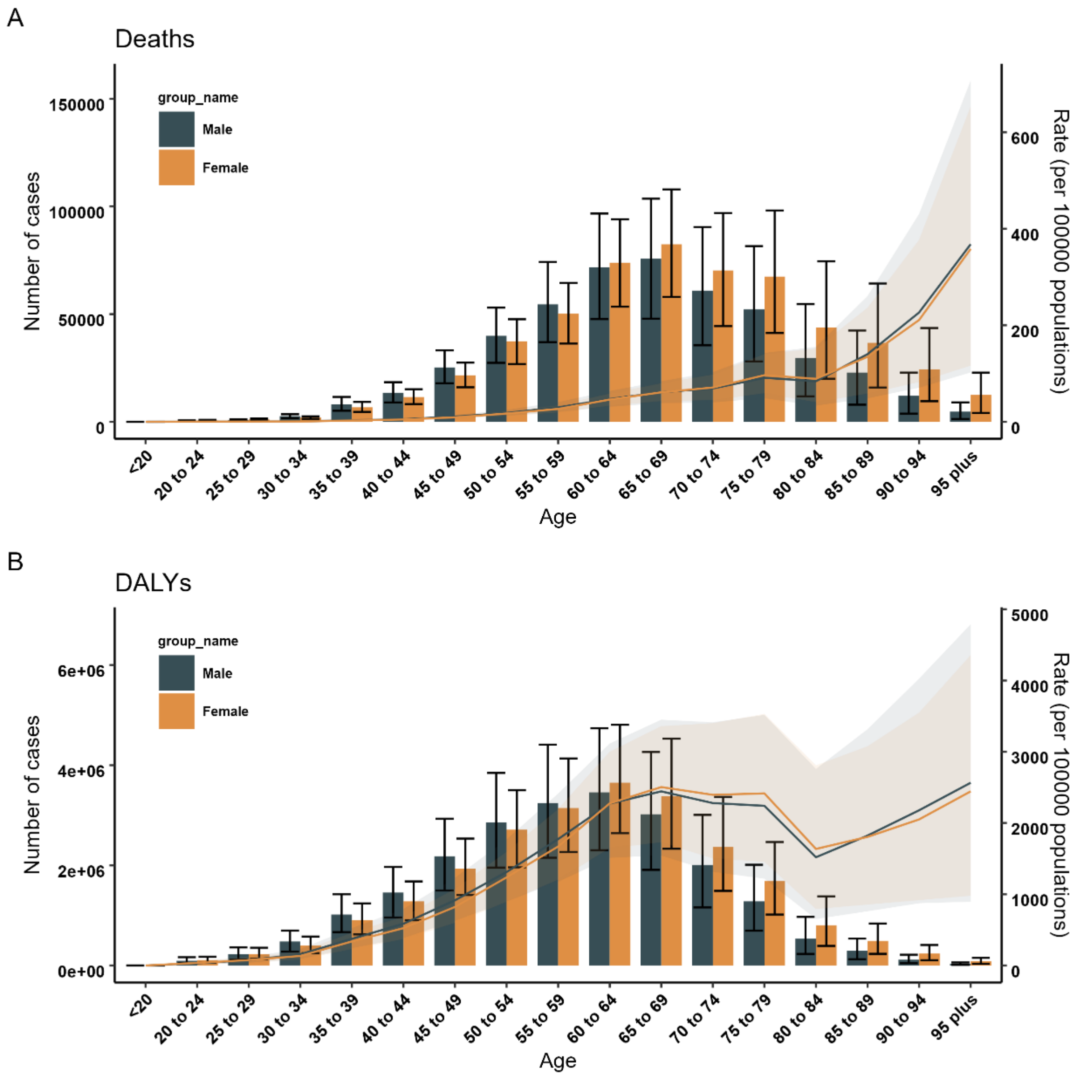

Supplementary Figure 2. Age-specific numbers and rates of deaths and DALYs for diabetes and kidney diseases due to high body mass index by sex, in 2019.

(A) Deaths. (B) DALYs. DALY, disability-adjusted life year.

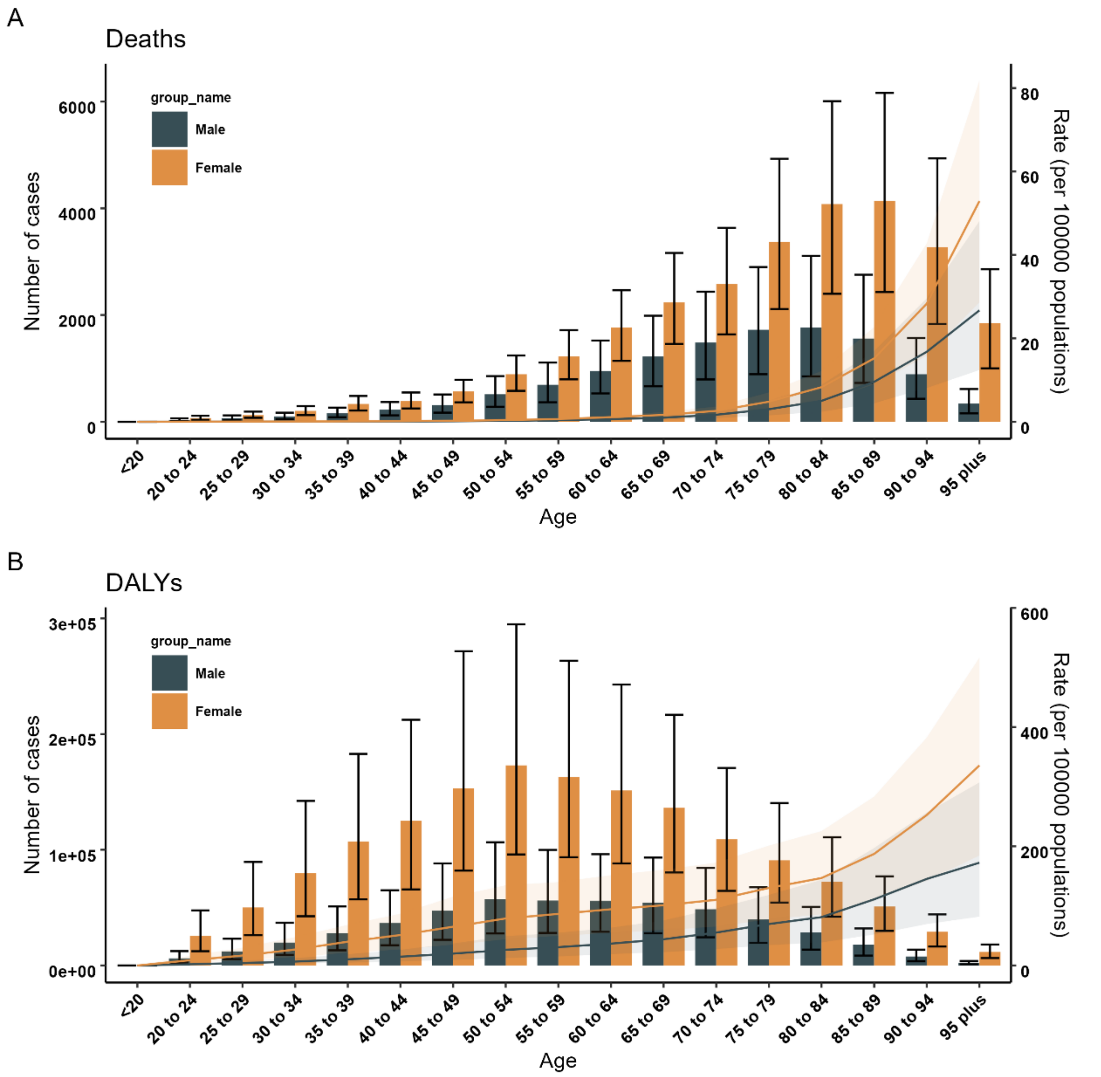

Supplementary Figure 3. Age-specific numbers and rates of deaths and DALYs for digestive diseases due to high body mass index by sex, in 2019.

(A) Deaths. (B) DALYs. DALY, disability-adjusted life year.

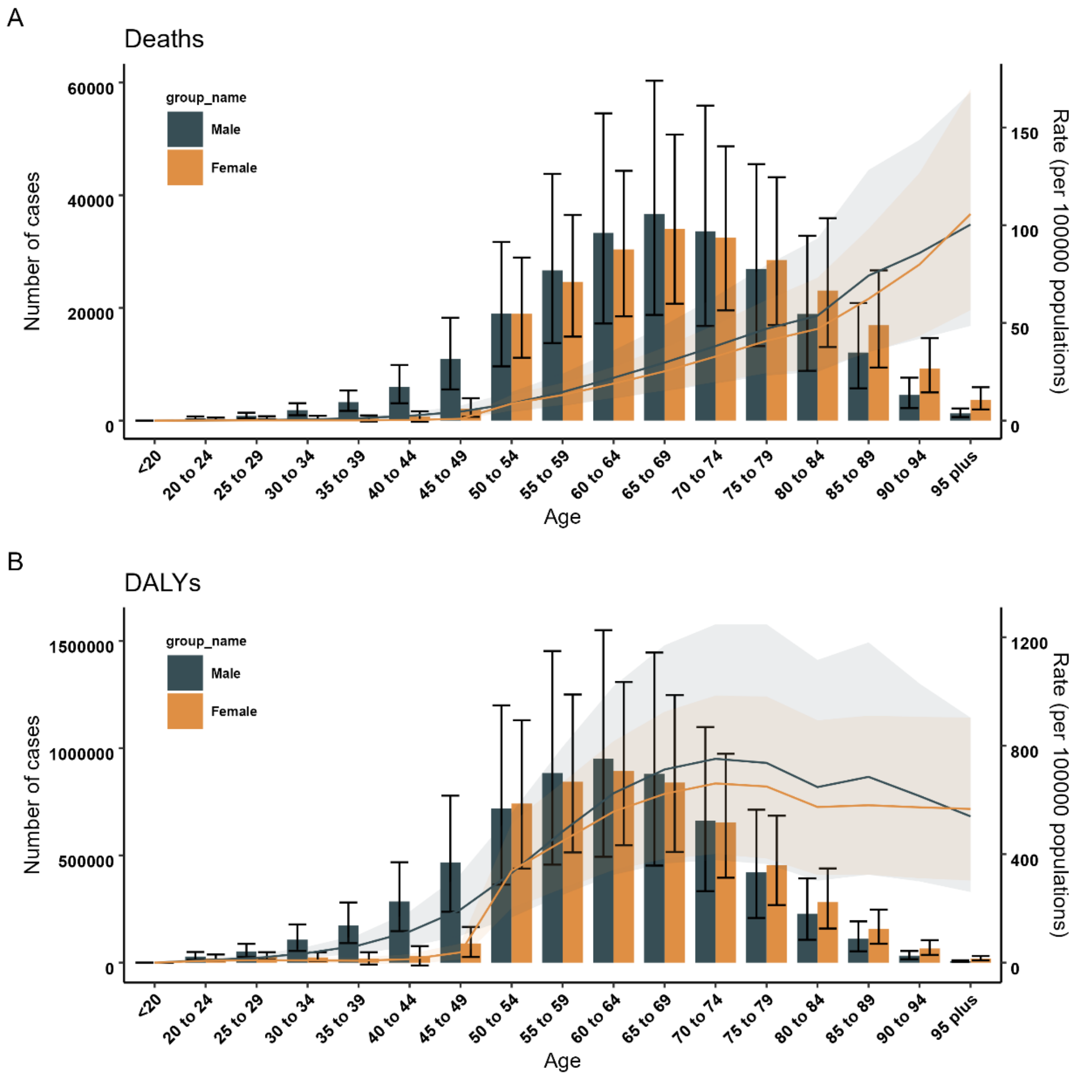

Supplementary Figure 4. Age-specific numbers and rates of deaths and DALYs for neoplasms due to high body mass index by sex, in 2019.

(A) Deaths. (B) DALYs. DALY, disability-adjusted life year.

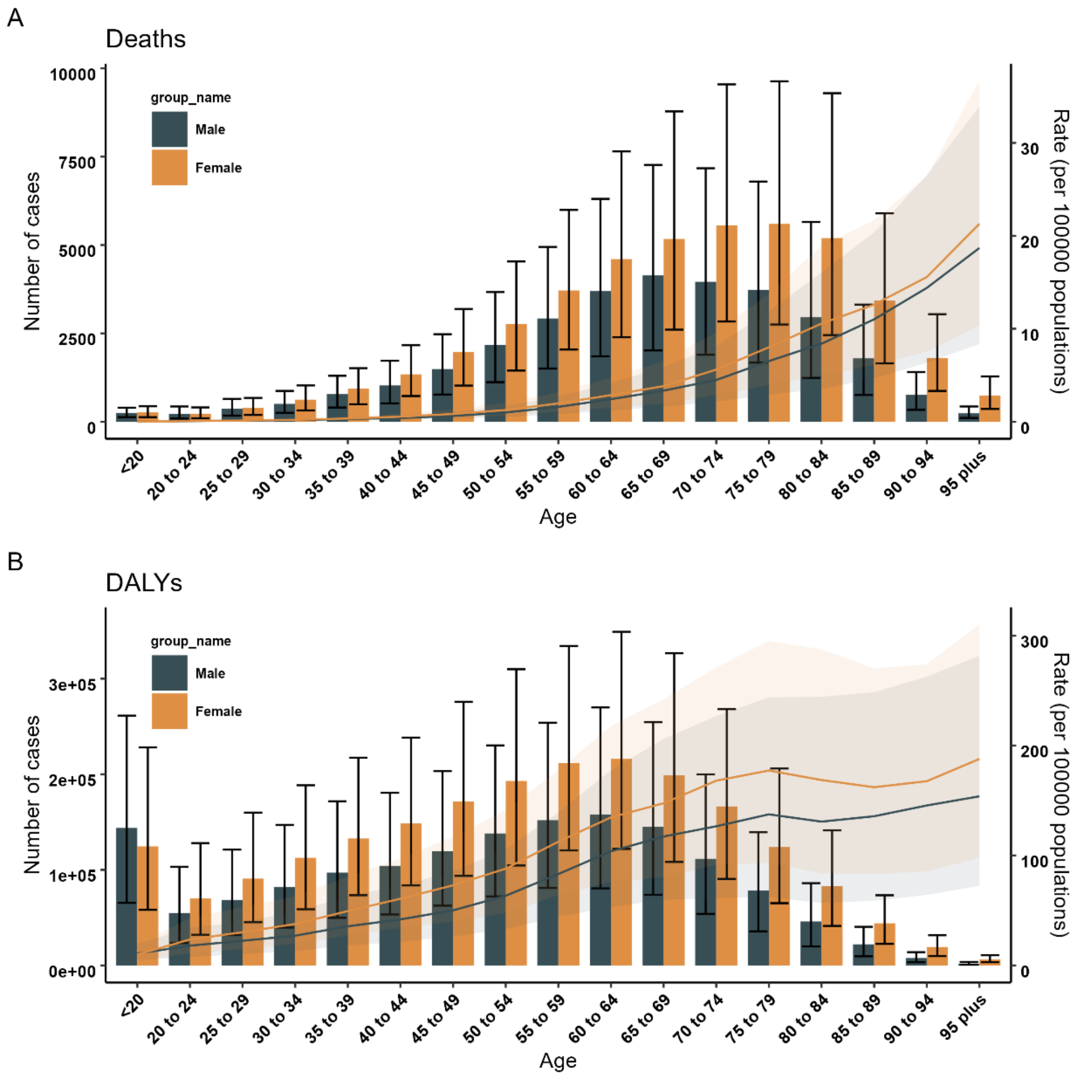

Supplementary Figure 5. Age-specific numbers and rates of deaths and DALYs for chronic respiratory diseases due to high body mass index by sex, in 2019.

(A) Deaths. (B) DALYs. DALY, disability-adjusted life year.

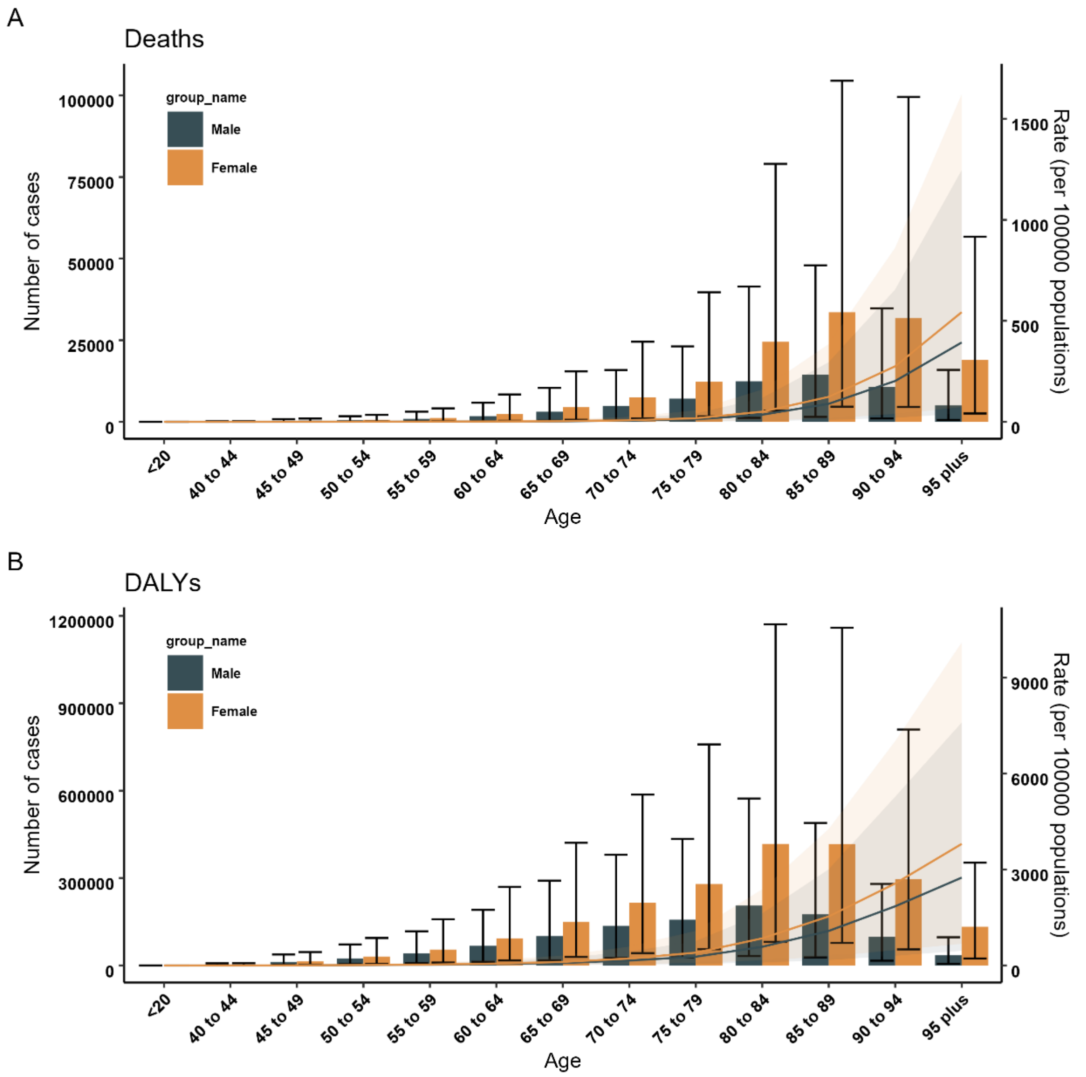

Supplementary Figure 6. Age-specific numbers and rates of deaths and DALYs for neurological disorders due to high body mass index by sex, in 2019.

(A) Deaths. (B) DALYs. DALY, disability-adjusted life year.

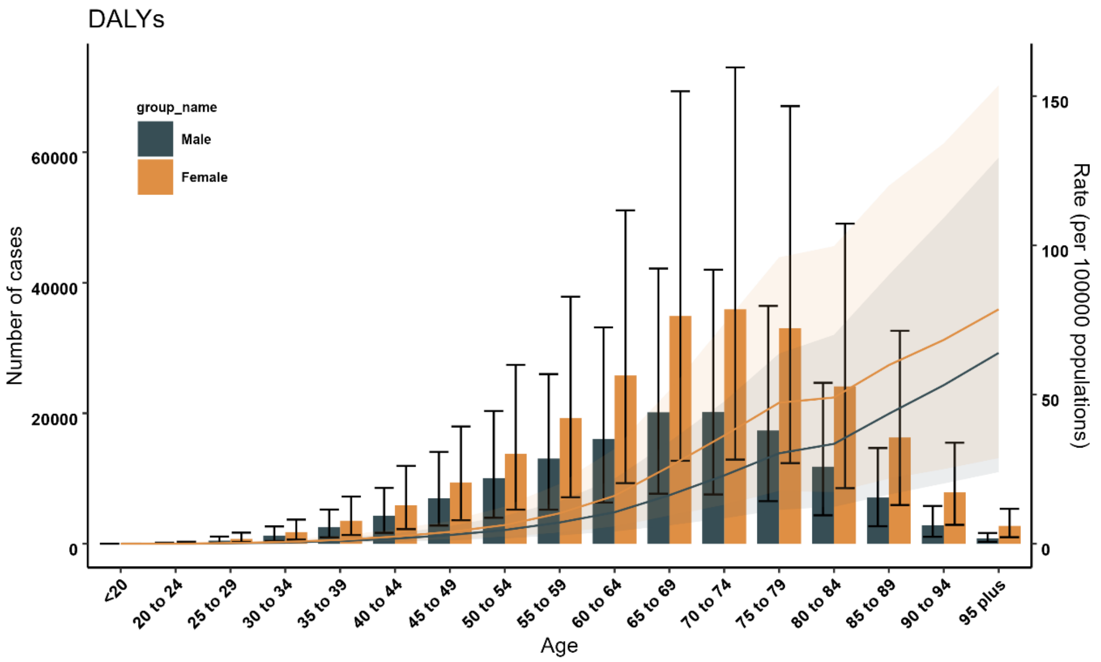

Supplementary Figure 7. Age-specific numbers and rates of DALYs for sense organ diseases due to high body mass index by sex, in 2019.

DALY, disability-adjusted life year.

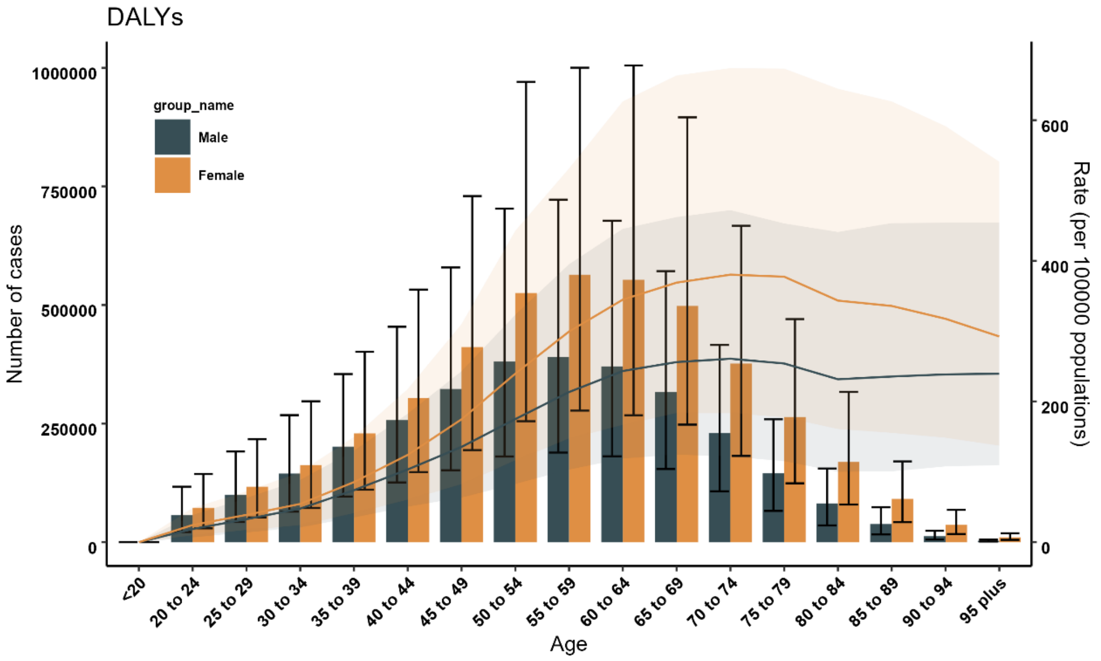

Supplementary Figure 8. Age-specific numbers and rates of DALYs for musculoskeletal disorders due to high body mass index by sex, in 2019.

DALY, disability-adjusted life year.

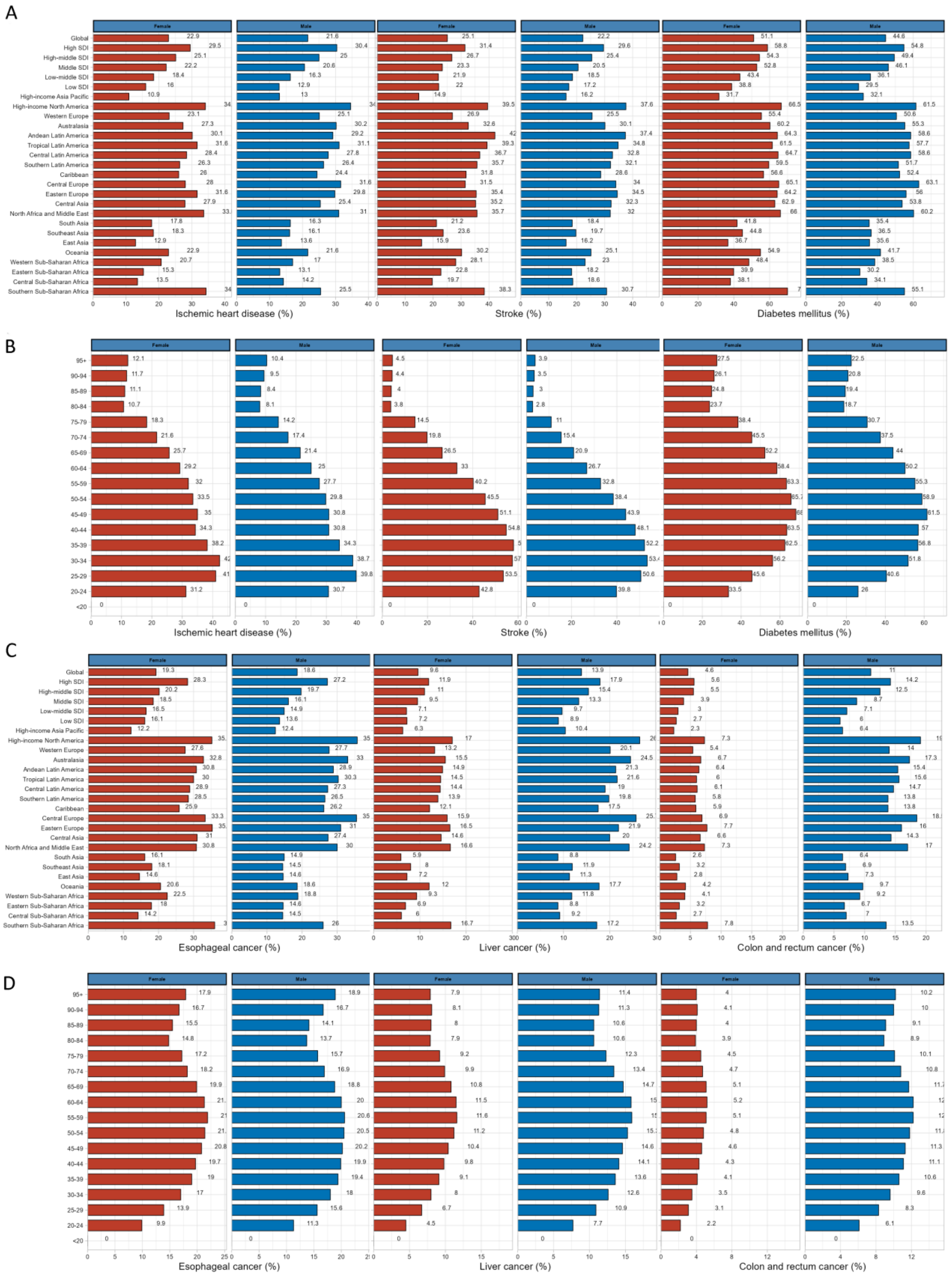

Supplementary Figure 9 Fraction of ischemic heart disease, stroke, and diabetes mellitus age-standardized DALYs associated with High BMI by region and by age group for women and men in 2019.

(A) By region for the 3 leading GBD level 3 of High BMI. (B) By age group for the 3 leading GBD level of High BMI. (C) By region for the 3 leading Cancer due to High BMI. (D) By age group for the 3 leading Cancer due to High BMI.

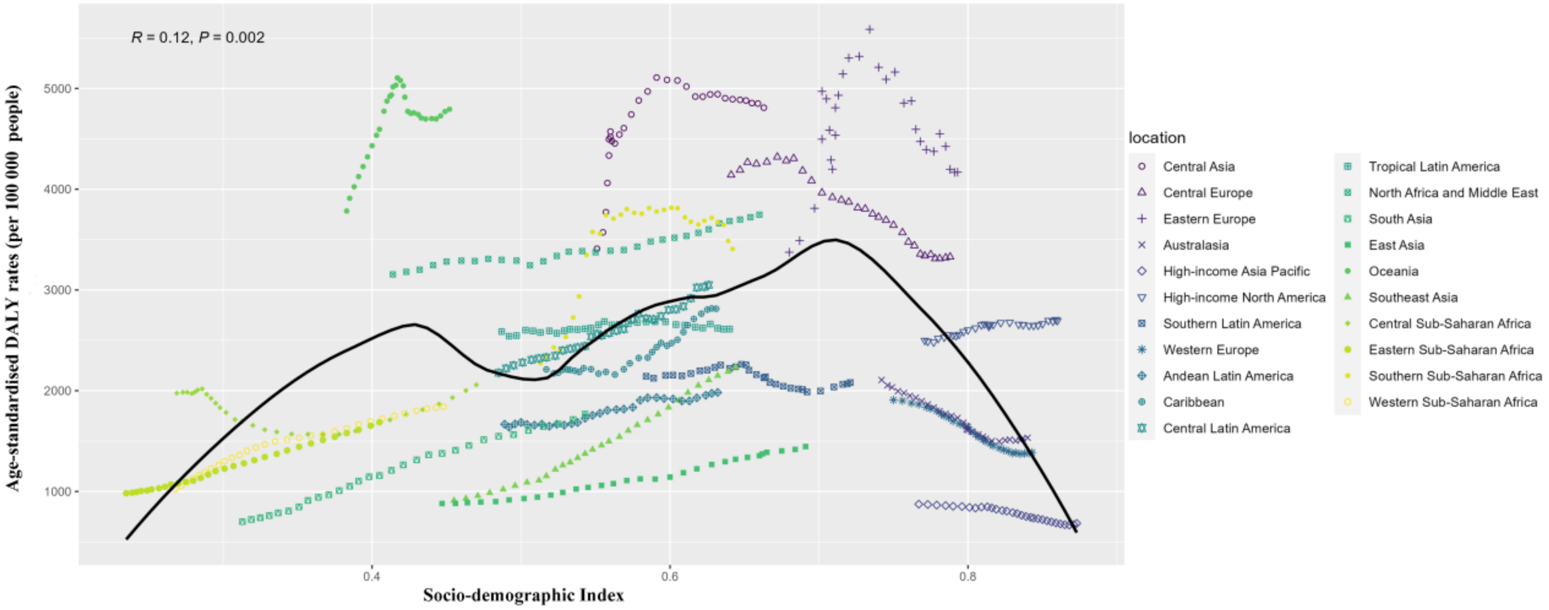

Supplementary Figure 10. Age-standardized DALY rates associated with High BMI across 21 GBD regions by socio-demographic index for both sexes from 1990 to 2019.

For each region, point from left to right depict estimates from each year from 1990 to 2019. DALY, disability-adjusted life year; GBD, Global Burden of Disease Study.

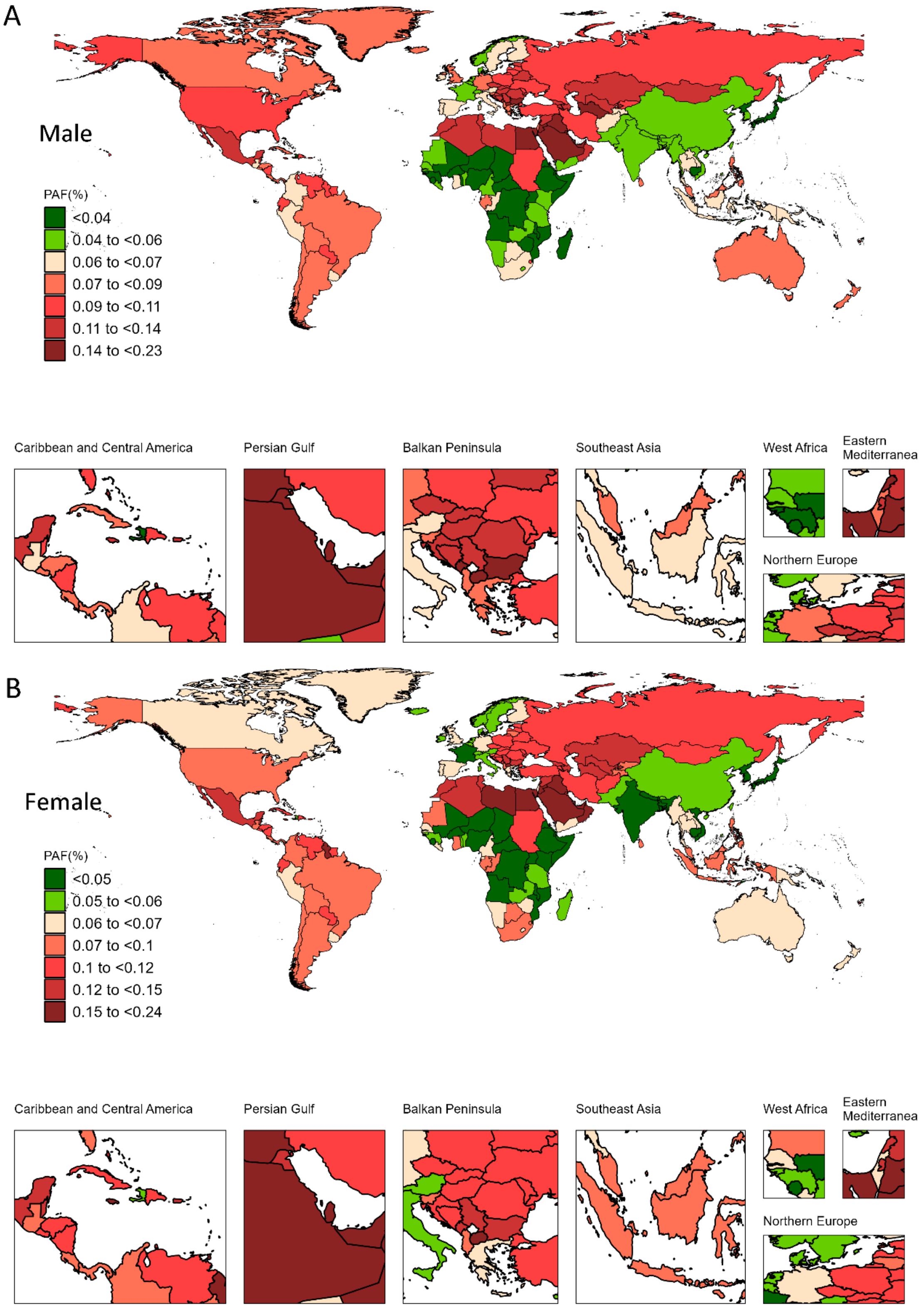

Supplementary Figure 11. PAF of new disease cases in 2019 caused by high BMI in men and women, by 204 countries and territories.

PAF=population attributable fraction.

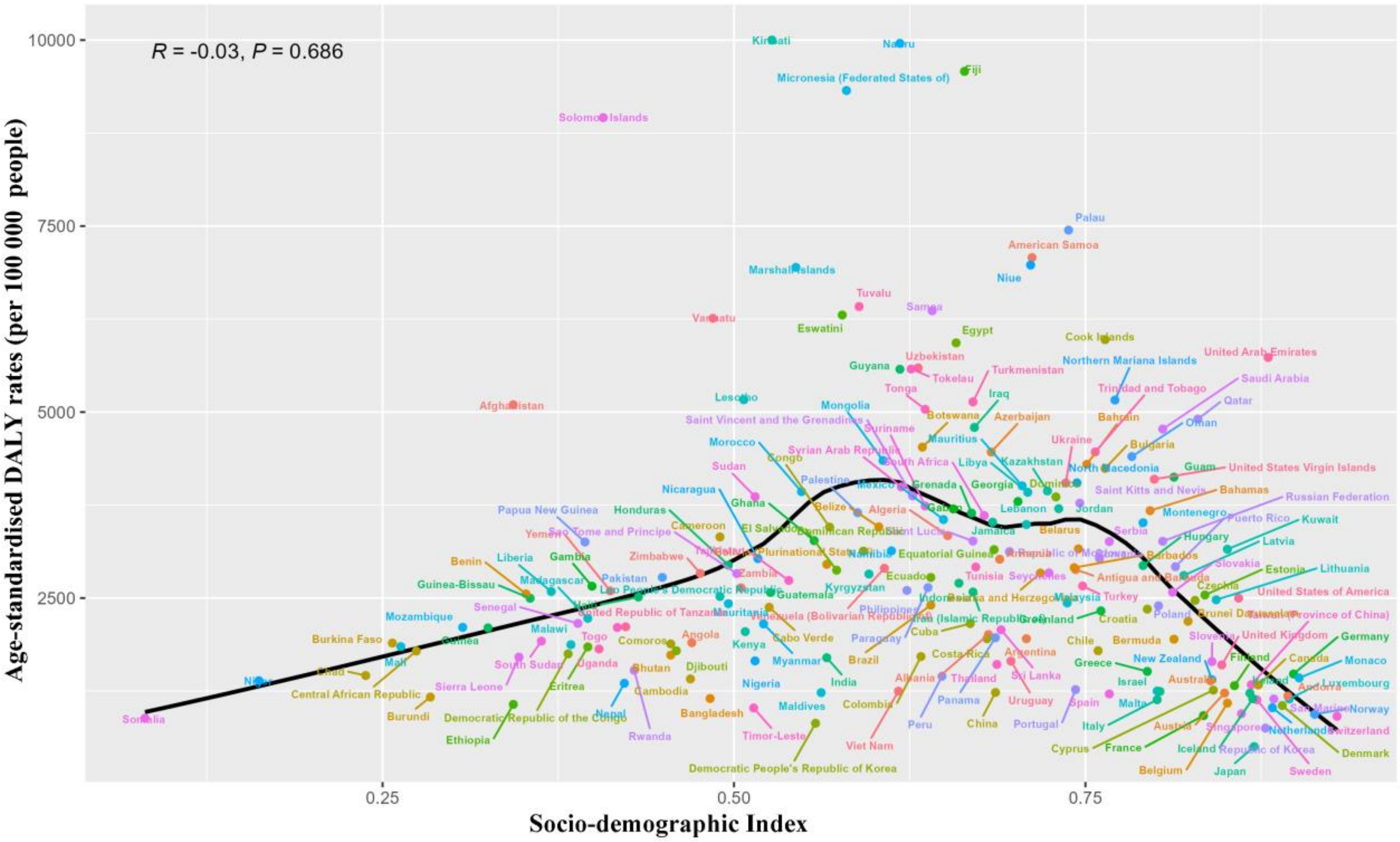

Supplementary Figure 12. Age-standardized DALY rates associated with High BMI across 204 countries and territories by Socio-demographic Index for both sexes, in 2019.

DALY, disability-adjusted life year.

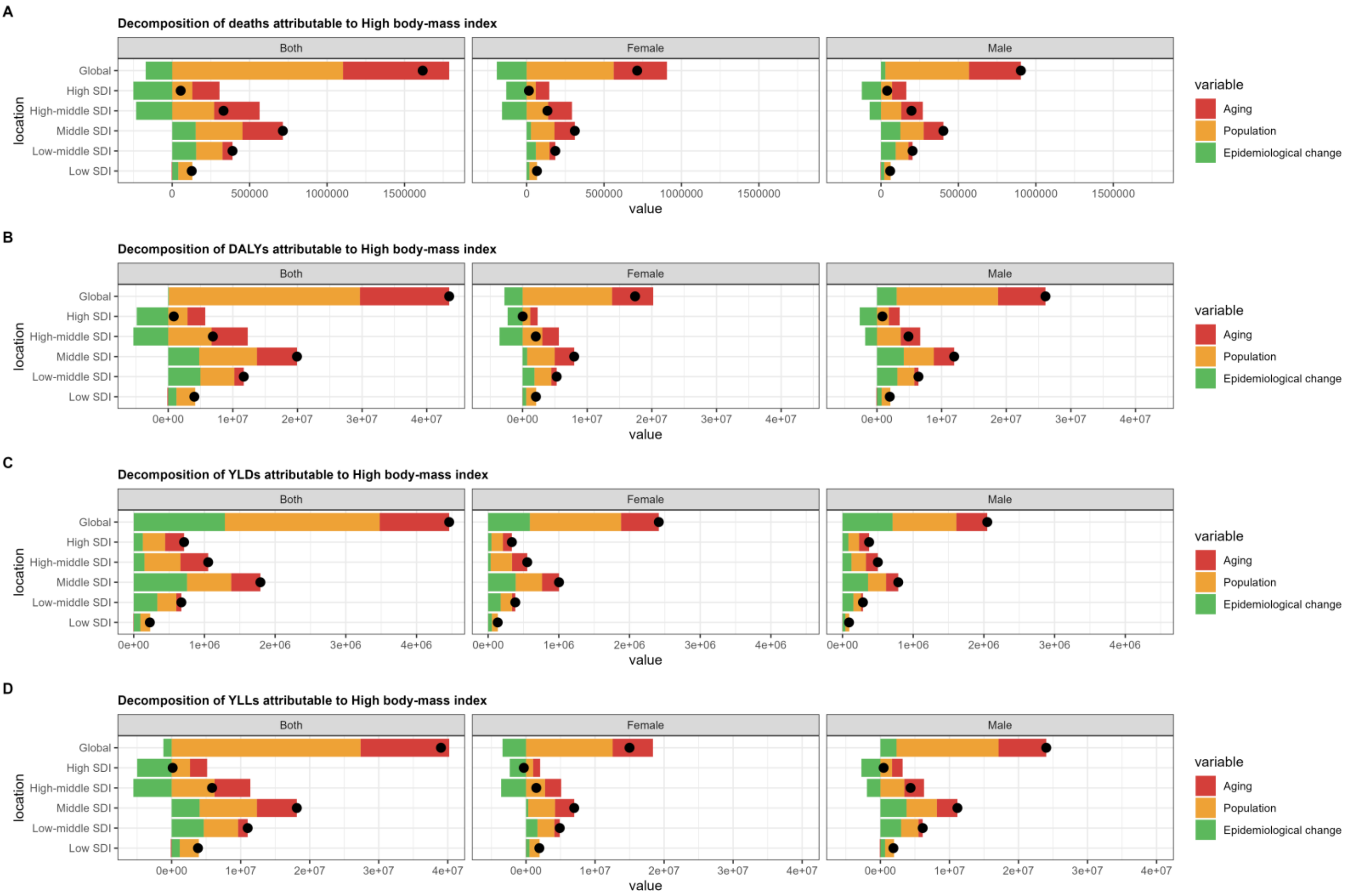

Supplementary Figure 13. Decomposition of deaths (A), DALYs (B), YLDs (C), and YLLs (D) in cardiovascular disease associated with high BMI in global and SDI regions, 1990 - 2019.

Legend: Percent change in risk-attributable deaths, DALYs, YLDs, and YLLs. Results are shown for all causes combined. The black dot shows total percentage change. The risk-deleted DALY rate is the expected DALY rate if the exposure level for high BMI was reduced to the theoretical minimum risk exposure level. DALYs, disability-adjusted life-years; YLDs, Years Lived with Disability; YLLs, Years of Life Lost; BMI, body mass index.

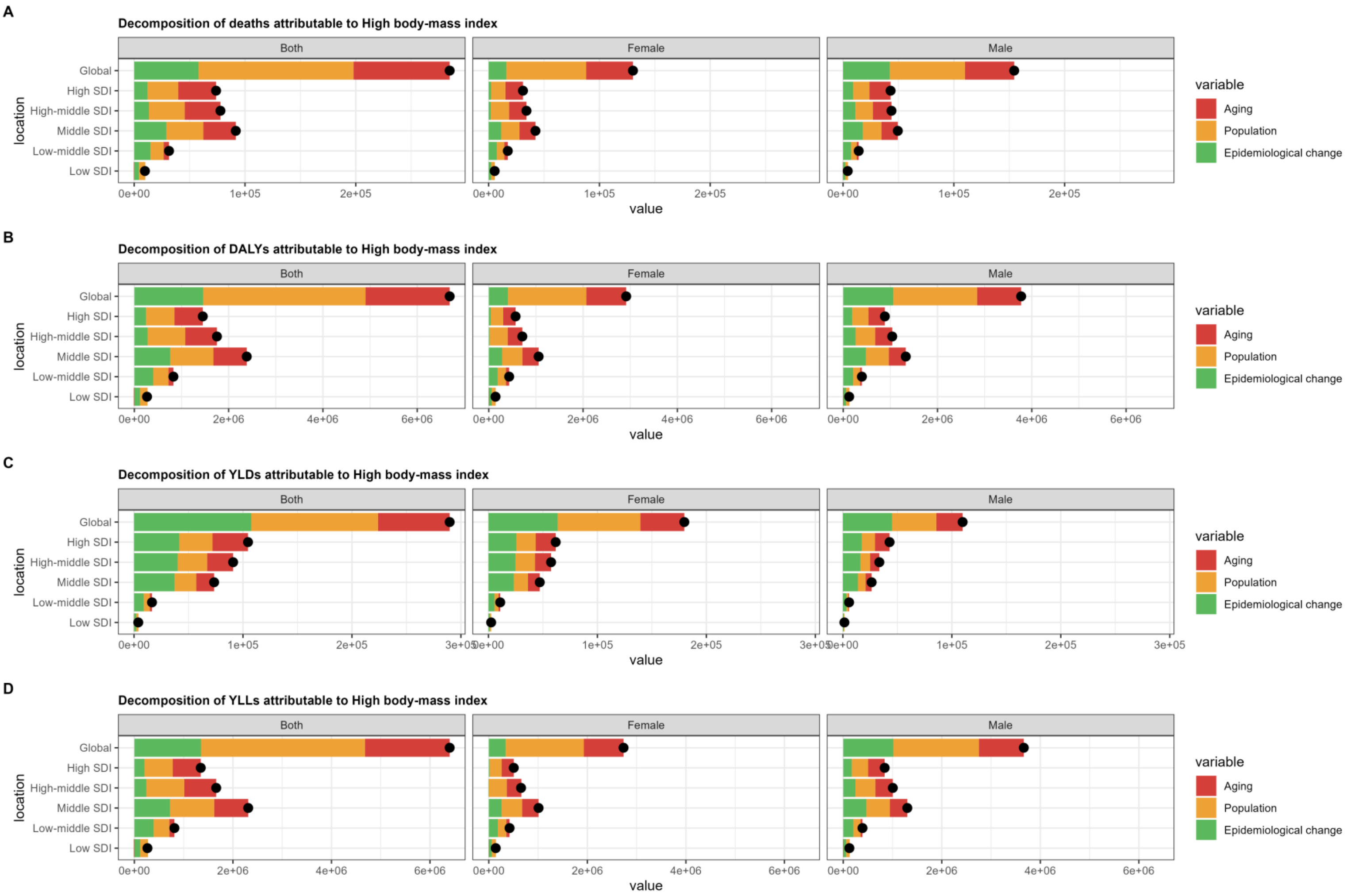

Supplementary Figure 14. Decomposition of deaths (A), DALYs (B), YLDs (C), and YLLs (D) in Neoplasms associated with high BMI in global and SDI regions, 1990 - 2019.

Legend: Percent change in risk-attributable deaths, DALYs, YLDs, and YLLs. Results are shown for all causes combined. The black dot shows total percentage change. The risk-deleted DALY rate is the expected DALY rate if the exposure level for high BMI was reduced to the theoretical minimum risk exposure level. DALYs, disability-adjusted life-years; YLDs, Years Lived with Disability; YLLs, Years of Life Lost; BMI, body mass index.

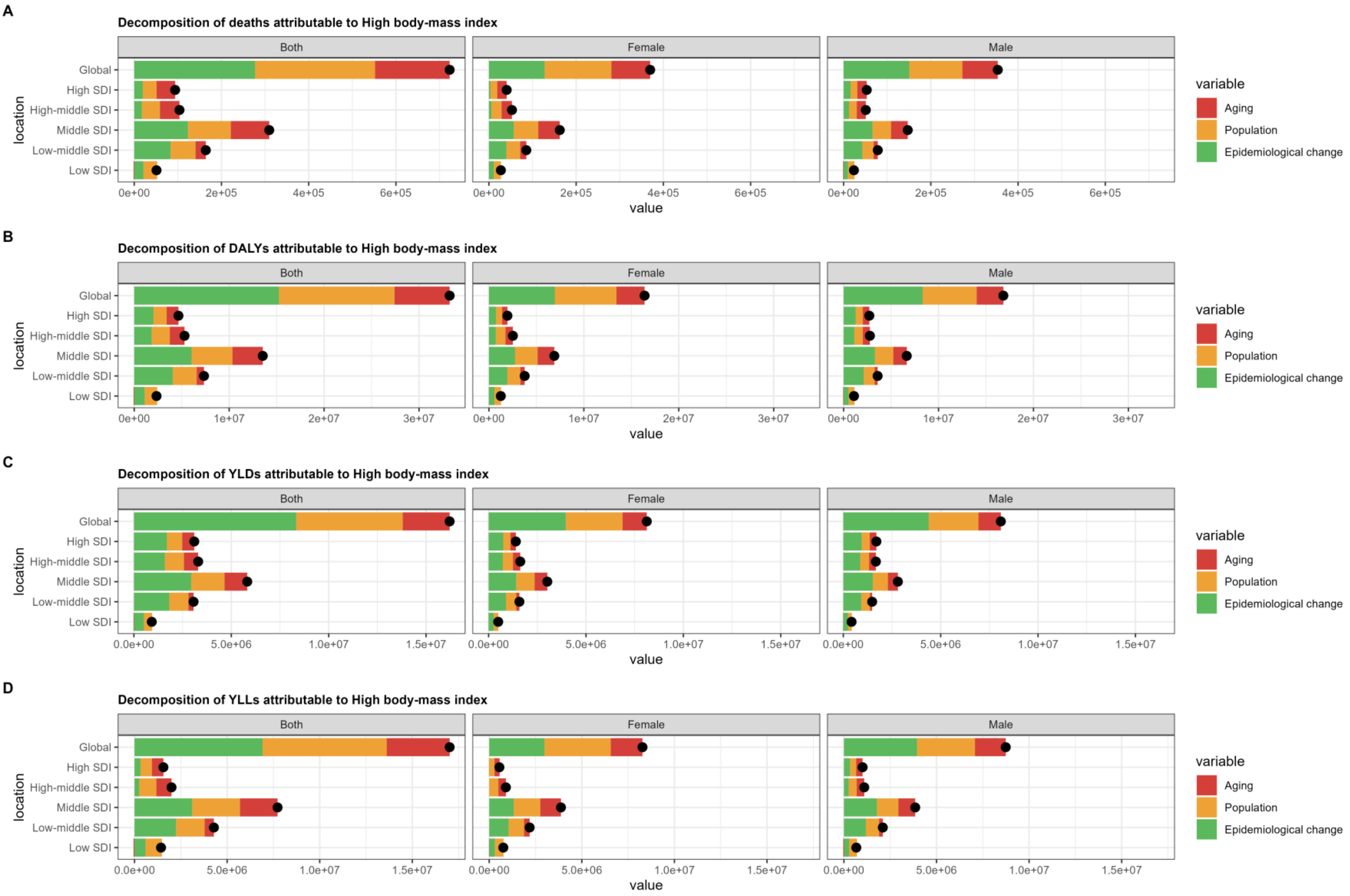

Supplementary Figure 15. Decomposition of deaths (A), DALYs (B), YLDs (C), and YLLs (D) in Diabetes and kidney diseases associated with high BMI in global and SDI regions, 1990 - 2019.

Legend: Percent change in risk-attributable deaths, DALYs, YLDs, and YLLs. Results are shown for all causes combined. The black dot shows total percentage change. The risk-deleted DALY rate is the expected DALY rate if the exposure level for high BMI was reduced to the theoretical minimum risk exposure level. DALYs, disability-adjusted life-years; YLDs, Years Lived with Disability; YLLs, Years of Life Lost; BMI, body mass index.

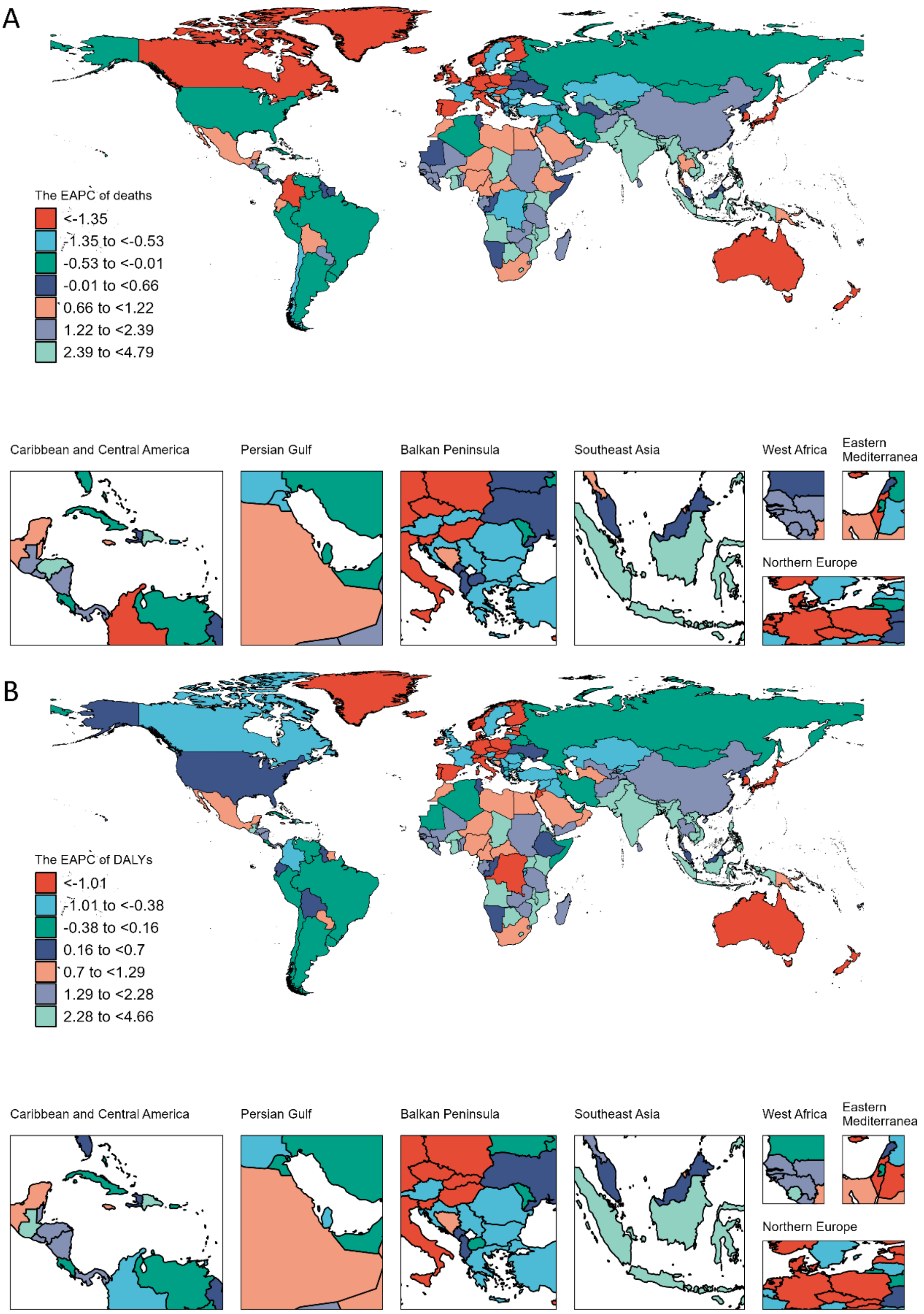

Supplementary Figure 16. The EAPC of deaths and DALYs rates associated with high body mass index for both sexes combined in 2019.

(A) Deaths. (B) DALYs.

Supplementary Table 1. Cause related with high BMI that contributed to deaths burden and DALYs in GBD 2019 in global.

Cause of deaths		Cause of deaths	
**Grade 2**	**Grade 3**	**Grade 2**	**Grade 3**
**Neoplasms**		**Neoplasms**	
	Thyroid cancer		Thyroid cancer
	Uterine cancer		Uterine cancer
	Ovarian cancer		Ovarian cancer
	Liver cancer		Liver cancer
	Colon and rectum cancer		Colon and rectum cancer
	Kidney cancer		Kidney cancer
	Breast cancer		Breast cancer
	Gallbladder and biliary tract cancer		Gallbladder and biliary tract cancer
	Pancreatic cancer		Pancreatic cancer
	Esophageal cancer		Esophageal cancer
	Leukemia		Leukemia
	Non-Hodgkin lymphoma		Non-Hodgkin lymphoma
	Multiple myeloma		Multiple myeloma
**Cardiovascular diseases**		**Cardiovascular diseases**	
	Stroke		Stroke
	Ischemic heart disease		Ischemic heart disease
	Hypertensive heart disease		Hypertensive heart disease
	Atrial fibrillation and flutter		Atrial fibrillation and flutter
**Chronic respiratory diseases**		**Chronic respiratory diseases**	
	Asthma		Asthma
**Digestive diseases**		**Digestive diseases**	
	Gallbladder and biliary diseases		Gallbladder and biliary diseases
**Neurological disorders**		**Neurological disorders**	
	Alzheimer's disease and other dementias		Alzheimer's disease and other dementias
**Diabetes and kidney diseases**		**Diabetes and kidney diseases**	
	Diabetes mellitus		Diabetes mellitus
	Chronic kidney disease		Chronic kidney disease
		**Sense organ diseases**	
			Blindness and vision loss
		**Musculoskeletal disorders**	
			Osteoarthtitis
			Low back pain
			Gout

BMI, body mass index; DALYs, disability-adjusted life-years

Supplementary Table 2. Global deaths and DALYs attributable to High BMI for neoplasm in 21 super regions and SDI regions in 2019 and percentage change from 1990 to 2019

Cause of death or DALYs	Deaths				DALYs			
	2019 age-standarized rate per 100,000 people	percentage change in age-standardized rate, 1990-2019	2019 age-standarized PAF	percentage change in age-standardized PAF,1990 to 2019	2019 age-standarized rate per 100,000 people	percentage change in age-standardized rate, 1990-2019	2019 age-standarized PAF	percentage change in age-standardized PAF, 1990 to 2019

**Global**	5.69 (3.21,8.83)	0.22 (0.07,0.43)	0.05 (0.03,0.07)	0.44 (0.28,0.68)	133.93 (76.19,206.81)	0.22 (0.05,0.46)	0.0437 (0.0252,0.0673)	0.52 (0.33,0.82)
**Gender**								
Male	6.29 (3.17,10.4)	0.35 (0.14,0.71)	0.04 (0.02,0.07)	0.62 (0.41,1.01)	150.73 (77.13,247.49)	0.33 (0.09,0.71)	0.04 (0.02,0.07)	0.68 (0.43,1.14)
Female	5.15 (3.1,7.73)	0.11 (-0.01,0.28)	0.05 (0.03,0.08)	0.31 (0.18,0.49)	117.76 (71.34,175)	0.12 (-0.02,0.31)	0.05 (0.03,0.07)	0.38 (0.23,0.61)
**Social-demograghic index**								
High SDI	7.53 (4.43,11.14)	0.12 (0.03,0.27)	0.06 (0.03,0.08)	0.4 (0.3,0.59)	174.68 (104.29,254.86)	0.11 (0.02,0.28)	0.0575 (0.0344,0.0838)	0.49 (0.37,0.71)
High-middle SDI	6.89 (3.93,10.61)	0.16 (0.03,0.33)	0.05 (0.03,0.08)	0.43 (0.29,0.61)	164.54 (94.43,251.91)	0.13 (-0.01,0.31)	0.0504 (0.0292,0.0761)	0.51 (0.35,0.73)
Middle SDI	5.03 (2.64,8.24)	0.55 (0.16,1.39)	0.04 (0.02,0.07)	0.74 (0.34,1.64)	126.04 (66.81,202.79)	0.5 (0.11,1.35)	0.0419 (0.0227,0.0667)	0.84 (0.39,1.83)
Low-middle SDI	2.92 (1.56,4.72)	1.16 (0.61,2.35)	0.03 (0.02,0.05)	1.13 (0.64,2.28)	72.76 (39.06,116.03)	1.15 (0.6,2.36)	0.0269 (0.0147,0.0431)	1.22 (0.69,2.41)
Low SDI	2.56 (1.28,4.28)	0.82 (0.44,1.69)	0.03 (0.01,0.04)	0.84 (0.48,1.63)	64.16 (32.79,105.68)	0.81 (0.42,1.69)	0.0241 (0.0124,0.0396)	0.91 (0.52,1.76)
**Region**								
**Africa**								
Southern Sub-Saharan Africa	9.63 (5.94,13.87)	0.38 (0.18,0.7)	0.07 (0.04,0.1)	0.34 (0.19,0.62)	222.87 (139.07,321.94)	0.28 (0.09,0.63)	0.0671 (0.0416,0.0951)	0.32 (0.16,0.63)
North Africa and Middle East	6.25 (3.89,9.04)	0.57 (0.31,0.97)	0.06 (0.04,0.09)	0.61 (0.39,0.94)	150.07 (92.52,216.05)	0.5 (0.24,0.91)	0.0597 (0.0378,0.0842)	0.64 (0.4,1)
Eastern Sub-Saharan Africa	3.64 (1.76,6.36)	1.05 (0.52,2.37)	0.03 (0.02,0.06)	1.1 (0.6,2.34)	90.55 (44.39,157.37)	0.99 (0.45,2.31)	0.0313 (0.0159,0.0517)	1.2 (0.64,2.56)
Western Sub-Saharan Africa	3.27 (1.79,5.22)	1.22 (0.71,2.33)	0.03 (0.02,0.05)	0.93 (0.57,1.77)	77.14 (42.24,122.66)	1.13 (0.62,2.19)	0.0304 (0.0176,0.0477)	0.96 (0.59,1.79)
Central Sub-Saharan Africa	2.81 (1.27,5.08)	0.13 (-0.12,0.56)	0.03 (0.01,0.04)	0.25 (0.06,0.6)	69.09 (31.36,123.27)	0.11 (-0.15,0.58)	0.0252 (0.0123,0.0427)	0.27 (0.07,0.66)
**America**								
High-income North America	9.57 (5.98,13.38)	0.25 (0.13,0.45)	0.07 (0.04,0.1)	0.54 (0.39,0.79)	227.1 (144.19,312.57)	0.22 (0.1,0.43)	0.0706 (0.0447,0.0964)	0.61 (0.45,0.87)
Southern Latin America	9.37 (5.42,13.99)	0.21 (0.05,0.52)	0.06 (0.04,0.09)	0.42 (0.24,0.78)	208.44 (122.98,309.57)	0.17 (0.02,0.45)	0.0579 (0.0342,0.0864)	0.45 (0.26,0.8)
Tropical Latin America	6.59 (4.06,9.57)	0.42 (0.21,0.8)	0.06 (0.04,0.08)	0.64 (0.4,1.06)	156.41 (95.94,226.37)	0.38 (0.19,0.73)	0.0544 (0.0332,0.0785)	0.64 (0.41,1.04)
Caribbean	6.58 (3.96,9.76)	0.32 (0.1,0.63)	0.05 (0.03,0.07)	0.34 (0.19,0.57)	161.57 (98.11,238.3)	0.32 (0.08,0.64)	0.0478 (0.0295,0.0685)	0.36 (0.19,0.6)
Andean Latin America	6.33 (3.64,9.59)	0.44 (0.13,0.96)	0.05 (0.03,0.08)	0.6 (0.39,1)	151.58 (88.74,228.99)	0.36 (0.06,0.81)	0.0535 (0.0329,0.0757)	0.6 (0.39,0.95)
Central Latin America	5.18 (2.98,7.77)	0.3 (0.09,0.6)	0.05 (0.03,0.08)	0.49 (0.34,0.76)	125.77 (73.35,186.5)	0.3 (0.09,0.59)	0.05 (0.0297,0.0735)	0.5 (0.35,0.74)
**Asia**								
Central Asia	8.07 (4.9,11.89)	0.12 (-0.06,0.41)	0.07 (0.04,0.1)	0.3 (0.13,0.62)	198.99 (121.44,290.9)	0.09 (-0.09,0.37)	0.0614 (0.0378,0.0897)	0.37 (0.17,0.69)
East Asia	4.98 (2.03,9.21)	0.43 (-0.02,1.94)	0.04 (0.01,0.06)	0.75 (0.26,2.44)	127.85 (52.8,231.5)	0.37 (-0.07,1.83)	0.0373 (0.0162,0.0664)	0.87 (0.33,2.7)
Southeast Asia	4.35 (2.23,7.1)	1.63 (0.87,3.52)	0.04 (0.02,0.06)	1.56 (0.9,3.3)	115.76 (60.48,184.82)	1.51 (0.81,3.24)	0.0398 (0.0214,0.0626)	1.62 (0.94,3.35)

High-income Asia Pacific	3.49 (1.33,6.55)	-0.01 (-0.11,0.21)	0.03 (0.01,0.06)	0.31 (0.19,0.59)	79.08 (31.06,146.31)	-0.06 (-0.16,0.15)	0.0312 (0.0122,0.0571)	0.35 (0.22,0.65)
South Asia	2.21 (1.19,3.51)	1.69 (0.85,3.82)	0.02 (0.01,0.04)	1.69 (0.95,3.69)	54.9 (29.76,86.87)	1.76 (0.9,3.89)	0.0229 (0.0126,0.0366)	1.78 (1.02,3.76)
**Europe**								
Central Europe	10.96 (6.88,15.57)	0.23 (0.07,0.42)	0.07 (0.04,0.09)	0.32 (0.22,0.46)	256.17 (162.83,364.18)	0.2 (0.04,0.39)	0.063 (0.0405,0.088)	0.38 (0.28,0.52)
Eastern Europe	9.01 (5.7,12.75)	0.29 (0.13,0.49)	0.07 (0.04,0.1)	0.55 (0.39,0.73)	224.51 (141.55,317.34)	0.27 (0.11,0.46)	0.0634 (0.0409,0.0881)	0.6 (0.45,0.77)
Western Europe	7.56 (4.36,11.34)	0.07 (0,0.2)	0.05 (0.03,0.08)	0.34 (0.25,0.49)	164.83 (96.63,245.73)	0.04 (-0.04,0.17)	0.0524 (0.0305,0.0781)	0.37 (0.28,0.53)
**Oceania**								
Australasia	8.44 (5.19,11.95)	0.13 (0,0.34)	0.07 (0.04,0.09)	0.45 (0.31,0.72)	190.25 (117.88,266.86)	0.09 (-0.02,0.29)	0.065 (0.0402,0.0912)	0.49 (0.34,0.75)
Oceania	5.91 (2.7,9.87)	0.33 (0.08,0.68)	0.05 (0.02,0.08)	0.23 (0.1,0.44)	161.21 (76.85,268.83)	0.31 (0.05,0.67)	0.0489 (0.0243,0.0787)	0.22 (0.08,0.43)

DALY, disability-adjusted life year; PAF, population attributable fraction; SDI, social demograghic index.

Supplementary Table 3 Global deaths and DALYs attributable to High BMI for cardiovascular diseases in 21 super regions and SDI regions in 2019 and percentage change from 1990 to 2019

Cause of death or DALYs	Deaths				DALYs			
	2019 age-standarized rate per 100,000 people	percentage change in age-standardized rate, 1990-2019	2019 age-standarized PAF	percentage change in age-standardized PAF,1990 to 2019	2019 age-standarized rate per 100,000 people	percentage change in age-standardized rate, 1990-2019	2019 age-standarized PAF	percentage change in age-standardized PAF,1990 to 2019
Global	40.1 (24.72,58.49)	-0.07 (-0.18,0.09)	0.17 (0.1,0.24)	0.37 (0.23,0.6)	1045.37 (677.97,1461.55)	-0.01 (-0.13,0.19)	0.2149 (0.1399,0.2968)	0.45 (0.28,0.74)
**Gender**								
Male	44.09 (25.97,65.78)	0.02 (-0.13,0.26)	0.16 (0.09,0.23)	0.45 (0.28,0.77)	1200.69 (748.5,1705.06)	0.08 (-0.08,0.35)	0.21 (0.13,0.29)	0.52 (0.33,0.89)
Female	35.83 (22.91,51.61)	-0.14 (-0.24,0.01)	0.18 (0.11,0.25)	0.32 (0.19,0.52)	889.73 (599.73,1237.2)	-0.09 (-0.2,0.08)	0.23 (0.15,0.3)	0.39 (0.24,0.64)
**Social-demograghic index**								
High SDI	25.62 (16.32,35.84)	-0.4 (-0.46, -0.29)	0.2 (0.13,0.28)	0.28 (0.15,0.51)	680.92 (465.64,908.11)	-0.34 (-0.41, -0.21)	0.2691 (0.1851,0.3553)	0.34 (0.2,0.59)
High-middle SDI	48.84 (30.49,70.83)	-0.24 (-0.31, -0.14)	0.19 (0.12,0.26)	0.23 (0.14,0.38)	1170.77 (762.81,1609.96)	-0.23 (-0.3, -0.13)	0.2394 (0.1569,0.3283)	0.26 (0.16,0.41)
Middle SDI	43.75 (26.09,65.52)	0.28 (0.03,0.73)	0.16 (0.09,0.23)	0.67 (0.38,1.23)	1157.2 (733.55,1647.73)	0.29 (0.03,0.76)	0.2108 (0.1349,0.2941)	0.76 (0.45,1.37)

Low-middle SDI	38.27 (22.25,57.82)	0.71 (0.3,1.62)	0.13 (0.08,0.2)	1.05 (0.59,2.1)	1058.22 (640.64,1533.68)	0.76 (0.32,1.7)	0.1754 (0.1067,0.2521)	1.16 (0.66,2.28)
Low SDI	35.09 (19.24,54.71)	0.42 (0.14,1.06)	0.12 (0.07,0.19)	0.7 (0.39,1.44)	968.76 (548.57,1448.88)	0.42 (0.12,1.07)	0.1594 (0.0932,0.2345)	0.75 (0.43,1.51)
**Region**								
**Africa**								
North Africa and Middle East	92.93 (61.71,128.76)	0 (-0.15,0.21)	0.26 (0.17,0.35)	0.38 (0.23,0.63)	2279.02 (1548.68,3054.69)	-0.05 (-0.2,0.15)	0.3213 (0.2256,0.4181)	0.41 (0.26,0.65)
Southern Sub-Saharan Africa	63.14 (43.91,83.72)	0.21 (0.07,0.38)	0.26 (0.18,0.34)	0.21 (0.12,0.34)	1491.83 (1107.32,1896.31)	0.05 (-0.06,0.18)	0.3107 (0.232,0.3911)	0.16 (0.09,0.27)
Central Sub-Saharan Africa	41.77 (21.71,67.37)	0 (-0.21,0.36)	0.13 (0.07,0.2)	0.16 (0.03,0.42)	1088 (575.49,1691.84)	-0.02 (-0.24,0.36)	0.1658 (0.093,0.2473)	0.2 (0.06,0.48)
Western Sub-Saharan Africa	41.55 (24.76,61.45)	0.55 (0.15,1.37)	0.16 (0.1,0.23)	0.86 (0.51,1.71)	1109.89 (693.02,1587.22)	0.51 (0.12,1.29)	0.2101 (0.138,0.2906)	0.85 (0.51,1.66)
Eastern Sub-Saharan Africa	36.69 (20.46,56.62)	0.52 (0.13,1.45)	0.14 (0.07,0.21)	0.82 (0.4,1.94)	974.42 (566.87,1429.19)	0.45 (0.06,1.33)	0.1747 (0.1042,0.2529)	0.87 (0.44,1.99)
**America**								
Caribbean	48.72 (30.05,70.78)	-0.03 (-0.18,0.2)	0.2 (0.13,0.29)	0.33 (0.2,0.56)	1303.17 (833.59,1847.21)	-0.01 (-0.18,0.23)	0.252 (0.1669,0.3414)	0.31 (0.18,0.55)
Tropical Latin America	41.79 (28.06,56.77)	-0.28 (-0.38, -0.08)	0.24 (0.16,0.32)	0.44 (0.24,0.84)	1108.32 (777.85,1459.54)	-0.31 (-0.4, -0.12)	0.2941 (0.2079,0.3844)	0.37 (0.19,0.74)
High-income North America	37.05 (24.18,49.88)	-0.25 (-0.33, -0.12)	0.24 (0.16,0.33)	0.29 (0.16,0.53)	993.26 (689.41,1267.7)	-0.22 (-0.29, -0.08)	0.3206 (0.2236,0.4066)	0.3 (0.18,0.53)
Central Latin America	35.78 (21.77,52.04)	-0.09 (-0.24,0.13)	0.22 (0.14,0.31)	0.31 (0.19,0.56)	888.35 (567.74,1241.79)	-0.12 (-0.26,0.08)	0.2856 (0.1865,0.3878)	0.29 (0.18,0.5)
Southern Latin America	33.14 (19.93,48.16)	-0.28 (-0.39,-0.06)	0.2 (0.12,0.29)	0.36 (0.18,0.77)	793.29 (505.22,1091.48)	-0.32 (-0.41, -0.12)	0.2523 (0.1611,0.3453)	0.33 (0.16,0.72)
Andean Latin America	29.29 (18.29,42.63)	-0.18 (-0.36,0.11)	0.24 (0.15,0.33)	0.41 (0.23,0.78)	771.78 (508.74,1075.91)	-0.23 (-0.39,0.02)	0.3131 (0.2153,0.4087)	0.38 (0.22,0.68)
**Asia**								
Central Asia	129.86 (84.3,181.55)	0.32 (0.18,0.53)	0.23 (0.15,0.31)	0.19 (0.1,0.33)	3019.37 (2031.16,4049.42)	0.22 (0.08,0.42)	0.2831 (0.191,0.3784)	0.17 (0.09,0.32)
Southeast Asia	39.19 (22.46,59.01)	1.12 (0.5,2.87)	0.14 (0.08,0.2)	1.37 (0.71,3.26)	1189.13 (725.67,1709.49)	1.09 (0.48,2.7)	0.1941 (0.1205,0.2724)	1.39 (0.74,3.2)

South Asia	33.64 (19.29,50.7)	1.01 (0.4,2.52)	0.13 (0.07,0.19)	1.5 (0.83,3.33)	959.49 (563.81,1401.55)	1.08 (0.44,2.61)	0.1654 (0.0993,0.237)	1.6 (0.89,3.47)
East Asia	28.73 (12.44,50.72)	0.28 (-0.08,1.52)	0.11 (0.05,0.18)	0.77 (0.35,2.42)	738.68 (339.72,1239.76)	0.28 (-0.1,1.58)	0.1502 (0.0719,0.2446)	0.91 (0.43,2.73)
High-income Asia Pacific	6.59 (2.72,11.7)	-0.58 (-0.63, -0.46)	0.08 (0.03,0.15)	0.14 (0.01,0.45)	200.84 (88.11,337.26)	-0.51 (-0.57, -0.38)	0.1251 (0.0549,0.2071)	0.2 (0.07,0.52)
**Europe**								
Eastern Europe	109.18 (70.51,150.46)	0.05 (-0.08,0.22)	0.23 (0.15,0.32)	0.26 (0.13,0.43)	2574.92 (1745.2,3461.57)	0.06 (-0.08,0.24)	0.2859 (0.1971,0.3768)	0.21 (0.09,0.37)
Central Europe	71.92 (47.07,99.99)	-0.33 (-0.42, -0.22)	0.23 (0.15,0.32)	0.14 (0.06,0.27)	1599.37 (1099.73,2134.13)	-0.38 (-0.46,-0.28)	0.2967 (0.2083,0.3883)	0.13 (0.06,0.25)
Western Europe	21.35 (12.57,31.82)	-0.49 (-0.54, -0.41)	0.17 (0.1,0.25)	0.13 (0.02,0.3)	468.37 (294.52,660.2)	-0.51 (-0.55, -0.44)	0.2171 (0.1374,0.3027)	0.13 (0.05,0.28)
**Oceania**								
Australasia	20.7 (12.94,29.41)	-0.55 (-0.6, -0.46)	0.18 (0.11,0.26)	0.12 (0,0.34)	490.26 (332.19,660.62)	-0.54 (-0.59, -0.46)	0.2513 (0.1721,0.3342)	0.18 (0.06,0.39)
Oceania	69.27 (36.96,112.97)	0.1 (-0.08,0.38)	0.17 (0.1,0.26)	0.11 (0.03,0.29)	2164.64 (1225.31,3406.53)	0.1 (-0.09,0.38)	0.2237 (0.1316,0.3263)	0.12 (0.04,0.29)

DALY, disability-adjusted life year; PAF, population attributable fraction; SDI, social demograghic index.

Supplementary Table 4. Global deaths and DALYs attributable to High BMI for diabetes and kidney diseases in 21 super regions and SDI regions in 2019 and percentage change from 1990 to 2019

Cause of death or DALYs	Deaths				DALYs			
	2019 age-standarized rate per 100,000 people	percentage change in age-standardized rate, 1990-2019	2019 age-standarized PAF	percentage change in age-standardized PAF,1990 to 2019	2019 age-standarized rate per 100,000 people	percentage change in age-standardized rate, 1990-2019	2019 age-standarized PAF	percentage change in age-standardized PAF,1990 to 2019
**Global**	12.54 (8.54,17.03)	0.61 (0.41,0.93)	0.33 (0.23,0.45)	0.46 (0.29,0.74)	547.26 (385.1,728.24)	0.82 (0.57,1.17)	0.3969 (0.2907,0.5065)	0.57 (0.37,0.87)
**Gender**								
Male	12.69 (8.17,17.98)	0.79 (0.49,1.29)	0.3 (0.19,0.41)	0.6 (0.37,1.05)	553.33 (372.93,748.41)	1.01 (0.69,1.55)	0.37 (0.26,0.48)	0.69 (0.43,1.14)
Female	12.4 (8.86,16.37)	0.49 (0.3,0.75)	0.36 (0.26,0.48)	0.39 (0.24,0.61)	540.64 (393.68,709.75)	0.67 (0.47,0.95)	0.43 (0.33,0.53)	0.49 (0.33,0.73)
**Social-demograghic index**								

High SDI	8.61 (5.86,11.65)	0.18 (0.04,0.36)	0.39 (0.26,0.52)	0.18 (0.05,0.36)	475.51 (338.76,635.07)	0.5 (0.35,0.72)	0.5007 (0.3819,0.6108)	0.27 (0.16,0.44)
High-middle SDI	9.1 (6.2,12.38)	0.17 (0.05,0.33)	0.36 (0.25,0.49)	0.25 (0.14,0.4)	434.56 (303.02,586.08)	0.39 (0.25,0.57)	0.4459 (0.3279,0.5613)	0.36 (0.25,0.52)
Middle SDI	16.21 (11.1,21.7)	0.87 (0.56,1.35)	0.34 (0.24,0.45)	0.68 (0.44,1.09)	649.99 (462.98,857.51)	1.01 (0.67,1.52)	0.4077 (0.3024,0.5165)	0.77 (0.5,1.22)
Low-middle SDI	14.85 (9.64,20.62)	1.52 (0.87,2.86)	0.28 (0.18,0.38)	1.19 (0.66,2.34)	594.69 (395.21,812.98)	1.79 (1.08,3.26)	0.3328 (0.2285,0.4398)	1.33 (0.76,2.55)
Low SDI	13.69 (8.05,20.23)	0.73 (0.34,1.56)	0.23 (0.14,0.34)	0.76 (0.41,1.55)	513.15 (311.53,731.63)	0.94 (0.52,1.87)	0.2807 (0.1785,0.3893)	0.86 (0.49,1.73)
**Region**								
**Africa**								
Southern Sub-Saharan Africa	51.27 (39.13,64.41)	0.87 (0.65,1.14)	0.5 (0.39,0.62)	0.23 (0.14,0.36)	1539.39 (1217.07,1889.75)	0.74 (0.58,0.95)	0.5604 (0.4554,0.6577)	0.23 (0.15,0.34)
North Africa and Middle East	26.31 (18.68,34.74)	0.21 (0.02,0.46)	0.46 (0.33,0.59)	0.31 (0.19,0.5)	1010.19 (756.67,1310.19)	0.47 (0.27,0.7)	0.5394 (0.4196,0.6494)	0.35 (0.23,0.52)
Western Sub-Saharan Africa	20.04 (12.84,28.43)	0.89 (0.45,1.81)	0.3 (0.2,0.42)	0.8 (0.46,1.6)	637.75 (436.66,867.21)	0.96 (0.52,1.88)	0.3531 (0.2518,0.4604)	0.84 (0.49,1.62)
Central Sub-Saharan Africa	16.22 (8.83,24.87)	0.07 (-0.17,0.47)	0.25 (0.14,0.35)	0.22 (0.08,0.51)	605.89 (359.32,887.01)	0.23 (-0.01,0.61)	0.3038 (0.1896,0.4198)	0.31 (0.17,0.62)
Eastern Sub-Saharan Africa	15.15 (8.82,22.82)	0.74 (0.25,1.97)	0.23 (0.14,0.34)	0.97 (0.47,2.29)	503.18 (310.3,723.94)	0.84 (0.32,2.09)	0.2883 (0.1857,0.3966)	1.1 (0.55,2.5)
**America**								
Central Latin America	42.23 (28.8,56.19)	0.53 (0.32,0.8)	0.45 (0.31,0.58)	0.15 (0.07,0.29)	1630.26 (1170.82,2134)	0.52 (0.36,0.74)	0.5172 (0.3773,0.6424)	0.15 (0.07,0.27)
Andean Latin America	26.02 (17.32,36.23)	0.77 (0.4,1.37)	0.43 (0.3,0.56)	0.35 (0.18,0.67)	885.1 (635.49,1171.09)	0.75 (0.45,1.21)	0.506 (0.3742,0.6242)	0.38 (0.23,0.66)
Caribbean	25.19 (16.97,34.51)	0.22 (0.01,0.53)	0.4 (0.29,0.52)	0.22 (0.12,0.41)	1113.05 (788.13,1476.18)	0.38 (0.19,0.62)	0.4716 (0.3518,0.5824)	0.23 (0.14,0.4)
Tropical Latin America	21.86 (16.1,28.38)	0.29 (0.11,0.63)	0.45 (0.33,0.58)	0.42 (0.23,0.79)	844.2 (635.69,1077.73)	0.31 (0.13,0.63)	0.5278 (0.4109,0.6397)	0.41 (0.23,0.74)
Southern Latin America	16.92 (10.81,23.57)	0.36 (0.16,0.73)	0.39 (0.25,0.54)	0.3 (0.1,0.66)	622.67 (420.28,842.43)	0.56 (0.35,0.97)	0.4731 (0.3321,0.6046)	0.39 (0.22,0.75)
High-income North America	14.11 (9.89,18.41)	0.42 (0.26,0.63)	0.47 (0.33,0.6)	0.14 (0.01,0.3)	717.17 (535.13,930.77)	0.55 (0.43,0.73)	0.5739 (0.441,0.679)	0.17 (0.09,0.3)
**Asia**								

Central Asia	19.07 (13.81,24.5)	1.51 (1.17,1.97)	0.47 (0.34,0.59)	0.27 (0.16,0.44)	827.69 (601.5,1074.05)	1.25 (1.02,1.58)	0.5078 (0.389,0.6182)	0.29 (0.2,0.45)
Southeast Asia	18.76 (11.74,26.78)	1.65 (0.87,3.66)	0.27 (0.17,0.38)	1.46 (0.77,3.29)	722.01 (477.64,988.22)	1.76 (0.98,3.7)	0.3331 (0.2262,0.4388)	1.49 (0.82,3.2)
South Asia	13.1 (8.14,18.69)	1.86 (0.95,4.19)	0.26 (0.16,0.36)	1.56 (0.83,3.48)	568.79 (364.47,791.97)	2.23 (1.26,4.55)	0.3241 (0.2172,0.4304)	1.65 (0.91,3.49)
East Asia	4.49 (2.09,7.44)	0.76 (0.26,2.21)	0.21 (0.1,0.33)	0.95 (0.49,2.44)	244.27 (125.16,383.63)	1.05 (0.48,2.95)	0.2912 (0.158,0.4326)	1.29 (0.68,3.34)
High-income Asia Pacific	2.47 (1.05,4.38)	-0.25 (-0.37,-0.03)	0.18 (0.08,0.32)	0.19 (0.03,0.53)	170.31 (79.67,284.31)	0.15 (0,0.5)	0.2683 (0.1306,0.4107)	0.34 (0.19,0.72)
**Europe**								
Central Europe	10.15 (7.26,13.37)	0.03 (-0.12,0.19)	0.47 (0.35,0.6)	0.1 (0.02,0.2)	604.69 (437.13,811.12)	0.3 (0.17,0.45)	0.587 (0.4667,0.6929)	0.2 (0.13,0.3)
Eastern Europe	5.57 (4.07,7.18)	0.53 (0.33,0.77)	0.48 (0.36,0.6)	0.26 (0.15,0.39)	328.93 (240.5,433.91)	0.44 (0.32,0.6)	0.5239 (0.4124,0.6241)	0.25 (0.15,0.37)
Western Europe	6.4 (3.9,9.58)	-0.12 (-0.26,0.01)	0.34 (0.2,0.5)	0.04 (-0.12,0.19)	361.15 (239.91,513.5)	0.28 (0.14,0.46)	0.4775 (0.3439,0.6012)	0.19 (0.11,0.34)
**Oceania**								
Australasia	8.07 (5.18,11.33)	0.08 (-0.08,0.31)	0.41 (0.26,0.56)	0.13 (-0.01,0.35)	357.98 (257.24,484.59)	0.37 (0.21,0.62)	0.5185 (0.3926,0.6281)	0.21 (0.1,0.4)
Oceania	54.56 (33.71,80.73)	0.57 (0.22,1.05)	0.37 (0.24,0.51)	0.18 (0.08,0.4)	1993.6 (1306.55,2827.35)	0.61 (0.32,1.02)	0.4366 (0.3019,0.5729)	0.19 (0.09,0.37)

DALY, disability-adjusted life year; PAF, population attributable fraction; SDI, social demograghic index.
